# Supplementary material for: Ecological and biogeographic drivers of biodiversity cannot be resolved using clade age-richness data
Source: Nat Commun. 2021 May 19;12:2945. doi: 10.1038/s41467-021-23307-5 (PMC8134473; doi:10.1038/s41467-021-23307-5)
Supplement: Supplementary file 1 — Supplementary Information [file 41467_2021_23307_MOESM1_ESM.pdf]

# Supplementary Information for

## Ecological and biogeographic drivers of biodiversity cannot be resolved using clade age-richness data

**Daniel L. Rabosky and Roger B. J. Benson**

**Corresponding Author: Daniel L. Rabosky.**
**E-mail: drabosky@umich.edu**

### This PDF file includes:

Supplementary Note 1: Mathematical background: birth-death process
Supplementary Note 2: Supplementary methods
Supplementary Note 3: Stem clade ages and justification
Supplementary Figs. S1 to S11
Supplementary Tables S1 to S4
Supplementary References

## 1. Supplementary Note 1: Mathematical background: birth-death process

The present article is concerned with estimators of the net diversification rate of a lineage: this is simply the difference between the speciation rate ($\lambda$) and the extinction rate ($\mu$), or $r = \lambda - \mu$. We are concerned specifically with *point estimates* of this rate for a single clade, as is performed widely in the recent literature, with the resulting rate estimates used for subsequent downstream macroevolutionary or macroecological inference. We refer to this estimate as the ARR (age-richness-rate) estimate, as a special case of estimation under the constant-rate birth-death process for a single clade. We are not concerned with the adequacy of the constant-rate birth-death model more generally, but only the case where the parameters of the model are estimated from single data point (e.g., a single age-richness pair, $[n, t]$).

**Below, we prove that the likelihood of a given age-richness pair is exactly equal for processes with positive and zero net diversification.** The processes are therefore non-identifiable unless additional data are used to inform the rate estimates. We first derive the maximum likelihood estimator for the net diversification rate $r$, using the theory of birth-death processes (1–4). We then show that this result is identical to the widely-cited "method of moments" estimator (4). We then prove that all values of the relative extinction rate $\epsilon$ ($\mu/\lambda$ ) have identical probability at the maximum, on the interval $0 \leq \epsilon < 1$. We then show that this probability is identical for the special case where the net diversification rate is zero, which immediately implies that the ARR process is not identifiable. For the process to be identifiable, there must be information in the data that enables a researcher to distinguish between processes with $r > 0$ and $r = 0$. If the $r = 0$ process is equally consistent with the data, then it is not scientifically justifiable to estimate positive net diversification rates and to subsequently use those estimates for downstream comparative analyses, unless additional information is used to validate the assumption of nonzero net diversification.

### A. Maximum likelihood estimate of the net diversification rate (ARR).

Kendall (1) derived the probability density that a birth-death process would contain exactly $n$ lineages after some time $t$ from the start of the process. Consider the process parameterized in per-lineage rates of speciation ($\lambda$), extinction ($\mu$), net diversification ($r = \lambda - \mu$), and relative extinction ($\epsilon = \mu/\lambda$). For the process initiated with a single ancestral lineage, the probability of $n$ lineages, given $r$ and $\epsilon$, is

$$P_{n,t} = (1-\alpha)(1-\beta)\beta^{n-1} \quad [1]$$

where

$$\alpha = P_{0,t} = \frac{\epsilon(e^{rt}-1)}{(e^{rt}-\epsilon)} \quad [2]$$

and $\beta = (1/\epsilon)\alpha$. Given that $\alpha = P_{0,t}$, we immediately obtain the probability of $n$ lineages conditional on survival of the process to time $t$ by dividing through by $1 - P_{0,t}$, or

$$P_{n,t|n\geq 1} = (1-\beta)\beta^{n-1} \quad [3]$$

and where

$$\beta = \frac{\alpha}{\epsilon} = \frac{e^{rt}-1}{e^{rt}-\epsilon} \quad [4]$$

To derive the ML estimate of $r$, we take the derivative of the log-likelihood function with respect to $r$. Expanding out the log-likelihood function, we have:

$$logL = \log(1-\epsilon) - \log(e^{rt}-\epsilon) + (n-1)[\log(e^{rt}-1) - \log(e^{rt}-\epsilon)] \quad [5]$$

and taking the derivative with respect to $r$ gives

$$\frac{d}{dr}logL = \frac{-nte^{rt}}{e^{rt}-\epsilon} + (n-1)\frac{te^{rt}}{e^{rt}-1} \quad [6]$$

To simplify this expression, we will use the substitution $Z = e^{rt}$ and set the expression equal to zero, given that $\frac{df(r)}{dr} = 0$ gives a local maximum or minimum of the likelihood function:

$$0 = \frac{-ntZ}{Z - \epsilon} + (n-1)\frac{tZ}{Z-1} \quad [7]$$

Solving this expression algebraically yields:

$$Z = e^{rt} = n(1-\epsilon) + \epsilon \quad [8]$$

And taking the logarithm of both sides and solving for $r$ gives the maximum likelihood estimator of net diversification rate, or

$$\hat{r} = \frac{1}{t}\log(n(1-\epsilon)+\epsilon) \quad [9]$$

It is easily seen that the point $\hat{r}$ represents a function maximum by inspecting $\frac{d^2 f(r)}{dr^2}$ . This estimator $\hat{r}$ is identical to the "method of moments" estimator given by Magallon and Sanderson (4).

**B. "Method of moments" estimator of the net diversification rate (ARR).** The method of moments estimator of net diversification is easily derived from the expected richness through time under the birth-death process. For the process beginning with a single ancestral lineage (*stem* estimator), we have:

$$N(t) = \frac{e^{rt} - \epsilon}{1 - \epsilon} \quad [10]$$

following Kendall (1), Bailey (2), and Raup (3); the above equation is equation A25 in (3). To derive the corresponding ARR estimator, we simply set $N(t)$ equal to the current diversity ($n$) and solve for $r$. This exercise gives:

$$e^{rt} = n(1-\epsilon) + \epsilon \quad [11]$$

or

$$\hat{r} = \frac{1}{t}\log(n(1-\epsilon)+\epsilon) \quad [12]$$

as given by Magallon and Sanderson (4) and which is identical to the maximum likelihood estimator derived in the previous subsection.

**C. For ARR, likelihood of the data is independent of relative extinction rate.** The "data" for the ARR estimator consist of a single bivariate datum: a value for clade age, and the corresponding species richness at that time. These are used to estimate the net diversification rate $r$ of the lineage, and the researcher typically chooses to perform inference assuming a particular level of relative extinction $\epsilon$. Note that an age-richness pair is one piece of data, not two: one cannot use the age alone, or the richness alone, to compute an estimate of $r$ under the birth-death model. For comparative analysis, researchers usually choose to compute ARR estimates for a set of clades assuming a common background $\epsilon$. Here, we show that the ARR estimates contain no information about relative extinction rate, and maximized likelihood of the data is identical for all values of $\epsilon$ ($0 \leq \epsilon \leq 1$.

To show that the likelihood of the data is independent of $\epsilon$ at the maximum, we will plug the maximum likelihood estimator of $r$ into the equation for $P_{n,t|n>0}$. If there is no information in the data that can be used to distinguish between different relative extinction rates, then the likelihood of the data at the maximum $\hat{r}$ should be independent of the value of $\epsilon$ selected for the analysis. As above, we have

$$P_{n,t|n>0} = (1-\beta)\beta^{n-1} \quad [13]$$

where

$$1 - \beta = \frac{1-\epsilon}{e^{rt} - \epsilon} \quad [14]$$

And observe that

$$e^{\hat{r}t} = e^{t \times \frac{1}{t}\log(n(1-\epsilon)+\epsilon} = n(1-\epsilon)+\epsilon \qquad [15]$$

Plugging this in to the master equation for $P_{n,t|n>0}$, we have:

$$P_{n,t|n>0} = \Big(\frac{1-\epsilon}{n(1-\epsilon)+\epsilon-\epsilon}\Big) \times \Big(\frac{n(1-\epsilon)+\epsilon-1}{n(1-\epsilon)+\epsilon-\epsilon}\Big)^{n-1} \qquad [16]$$

which, after algebraic reduction, gives:

$$P_{n,t|n>0} = \Big(\frac{1}{n}\Big) \times \Big(\frac{n-1}{n}\Big)^{n-1} \qquad [17]$$

And the probability of the data is thus independent of the relative extinction rate $\epsilon$ at the maximum, $\hat{r}$. This equivalence is not expected to hold for the unconditioned likelihood $P_{n,t}$, because the likelihood of observing any $n$ with $n > 0$ is a decreasing function of $\epsilon$. However, we have not repeated this exercise with the corresponding ARR estimator $\hat{r}$ for the unconditioned process.

For the special case of a clade with $n = 1$ species at time $t$, the probability in equation 17 is not defined for all $\epsilon$. The reason is that the maximum likelihood estimate of $r$ for $n = 1$ is 0, as can be seen by inspection from equation 12. Because $r = \lambda - \mu$, a value of $r = 0$ immediately implies that $\lambda = \mu$ and $\epsilon = \frac{\mu}{\lambda} = 1$. At the maximum, $r = 0$ and $\epsilon = 1$, and the estimator is identifiable, because it is restricted to a single point ($r = 0$, $\epsilon = 1$). At this point, the probability of the data is 1.

**D. The "balanced" speciation-extinction process and non-identifiability of ARR.** For the "balanced" diversification process, $\lambda = \mu$, $\epsilon = 1$, and $r = 0$. It does not make sense to compare net diversification rates for clades if $r = 0$. However, for a single datum, the likelihood of the data at the maximum (with $\epsilon = 1$) is exactly equal to the likelihood with $\epsilon < 1$. In the absence of any other data, the net diversification rate for a single datum is thus non-identifiable: the likelihood of the data with $r > 0$ is exactly equal to the likelihood of the data with $r = 0$. To prove this, we first derive the maximum likelihood estimator of the speciation rate $\lambda$, then substitute this estimator into the equation describing the probabilty of observing $n$ species, given $\lambda$, conditional on the process surviving to time $t$. This probability is denoted by $P_{n,t|\lambda=\mu,n>0}$. Following Kendall (1) (equation 17), we have the chance of extinction for the $r = 0$ process, as:

$$P_{0,t} = \frac{\lambda t}{1+\lambda t} \qquad [18]$$

and Bailey (2) (equation 8.53) gives the corresponding probability of observing $n$ species at time $t$:

$$P_{n,t} = \frac{(\lambda t)^{n-1}}{(1+\lambda t)^{n+1}} \qquad [19]$$

The conditioned likelihood $P_{n,t|\lambda=\mu,n>0}$ can be easily derived from this by noting that

$$1 - P_{0,t} = \frac{1}{(1+\lambda t)} \qquad [20]$$

And we have immediately

$$P_{n,t|n>0,\lambda=\mu} = \frac{P_{n,t}}{(1-P_{0,t})} = \frac{(\lambda t)^{n-1}}{(1+\lambda t)^{n+1}} \times (1+\lambda t) = \frac{(\lambda t)^{n-1}}{(1+\lambda t)^{n}} \qquad [21]$$

To derive the maximum likelihood estimate of the speciation rate under the $r = 0$ process, we note that the log likelihood of a given age-richness datum ($n$, $t$) is given by

$$\log P_{n,t|n>0,\lambda=\mu} = (n-1)\log(\lambda) + (n-1)\log(t) - n\log(1+\lambda t) \qquad [22]$$

Taking the derivative with respect to $\lambda$, we have

$$\frac{dP_{n,t|n>0,\lambda=\mu}}{d\lambda} = \frac{n-1}{\lambda} - \frac{nt}{1+\lambda t} \quad [23]$$

Setting the derivative equal to zero and solving for $\lambda$ gives

$$\hat{\lambda} = (n-1)/t \quad [24]$$

which is the maximum likelihood estimator of the speciation rate, $\hat{\lambda}$, under the conditioned $\lambda = \mu$ process. We can obtain the same estimator of $\hat{\lambda}$ using the "method of moments". We first note that the expected number of species under the conditioned process is given by

$$N(t) = 1 + \lambda t \quad [25]$$

as noted by Raup (3), equation A23. As before, we simply equate $N(t)$ with the observed richness $n$ and solve for $\lambda$, giving the same result as the maximum likelihood estimator derived above.

We now derive the probability of $n$ species under the balanced process at the maximum, $\hat{\lambda}$. Given the ML estimator of $\lambda$, we have immediately

$$P_{n,t|\hat{\lambda},n>0,\lambda=\mu} = \frac{\left(\hat{\lambda}\right)^{n-1}}{\left(1+\hat{\lambda}t\right)^{n}} = \frac{\left(\frac{n-1}{t} \times t\right)^{n-1}}{\left(1+\frac{(n-1)}{t} \times t\right)^{n}} = \frac{(n-1)^{n-1}}{n^{n}} \quad [26]$$

And a trivial rearrangement reveals this as a geometric probability

$$P_{n,t|\hat{r},n>0,\lambda=\mu} = \left(\frac{1}{n}\right) \times \left(\frac{n-1}{n}\right)^{n-1} \quad [27]$$

and this probability is equal to that derived for the process with $r > 0$ (equation 17). Thus, under the ARR process, the probability of observing a given age-richness pair $(n, t)$ is exactly equal under both $r = 0$ and $r > 0$ conditions. This probability of $n$ species given the maximum likelihood (ARR) estimates of $\hat{r}$ (for $r > 0$) or $\hat{\lambda}$ (for $r = 0$) is geometric with parameter $1/n$. There is therefore no information in the data that can be used to distinguish between the $r = 0$ process and the $r > 0$ process. They yield exactly the same probability of a given age-richness pair. **The ARR estimator, as used in practice, is therefore non-identifiably distinct from a process with zero net diversification.** With no further information, a researcher is equally justified in performing comparative analyses with the assumption of $r = 0$ and $r > 0$.

**E. Non-identifiability, part 2: maximum likelihood inference on *relative extinction* with constant net diversification across clades.** . In this section, we prove a related non-identifiability property of ARR estimators. Consider a set of clades for which researchers might compute ARR estimates under some assumed relative extinction fraction (e.g., $\epsilon = 0.9$). Just as there is a maximum likelihood estimate of the net diversification rate $r$ given some assumed relative extinction fraction $\epsilon$, there is equivalently a maximum likelihood estimate of $\epsilon$ given some assumed net diversification rate $r$. We will first derive the maximum likelihood estimate of $\epsilon$; we will then show that the probability of the data at the maximum is identical to that given in equation 17:

$$P_{n,t|\hat{\epsilon},n>0,r>0} = P_{n,t|\hat{r},n>0,0\leq\epsilon\leq1} \quad [28]$$

This equality challenges the fundamental logic that underlies comparative studies of clade diversification using the ARR estimator. The ARR framework assumes that clades vary in net diversification rate, typically with some fixed extinction fraction $\epsilon$. If there is no variation in diversification rate, then there is nothing

to correlate with clade-level traits, biogeography, or other factors. Yet, on first principles, one could just as easily assume that all clades have identical net diversification rates and compute maximum likelihood estimates of the relative extinction fraction for each clade. This scenario, where all clades have an identical value of $r$ (but vary in $\epsilon$), cannot be distinguished from the widely-assumed scenario whereby clades have constant $\epsilon$ (but vary in $r$).

To begin, note first the log-likelihood of a given age-richness datum, as given in equation 5, and with the substitution $Z = e^{rt}$:

$$logL = \log(1-\epsilon) - n\log(Z-\epsilon) + (n-1)\log(Z-1) \qquad [29]$$

Taking the derivative of the likelihood function with respect to $\epsilon$, we have

$$\frac{dL}{d\epsilon} = \frac{n}{Z-\epsilon} - \frac{1}{1-\epsilon} \qquad [30]$$

Setting $\frac{dL}{d\epsilon} = 0$ and solving for $\epsilon$ gives the maximum likelihood estimate of the extinction fraction:

$$\hat{\epsilon} = \frac{n - e^{rt}}{n-1} \qquad [31]$$

This estimator is identical to that which would be obtained by the method of moments. To verify, we note again that

$$N(t) = \frac{e^{rt} - \epsilon}{1-\epsilon} \qquad [32]$$

and the estimate of $\hat{\epsilon}$ follows from equating the mean $N(t)$ with an observation of clade diversity $n$; solving this expression algebraically yields a solution that is identical to equation 31. Note that at the MLE for $\epsilon$, we have immediately

$$\hat{\beta} = \frac{Z-1}{Z-\hat{\epsilon}} = \frac{Z-1}{Z - \frac{n-Z}{n-1}} = \frac{n-1}{n} \qquad [33]$$

where $\beta$ was previously defined in equation 4. Plugging this $\hat{\beta}$ into the survival-conditioned probability for $n$ species, we have:

$$P_{n,t|n\geq 1} = (1-\beta)\beta^{n-1} = \left(\frac{1}{n}\right) \times \left(\frac{n-1}{n}\right)^{n-1} \qquad [34]$$

which is identical to the probability of the data under $\hat{r}$, or equation 17.

Finally, we must compute the interval from which we can choose $r$, noting that similar constraints exist for $\epsilon$ (e.g., we cannot choose to compute $r$ under $\epsilon < 0$). Under the special case where $n = 1$, the maximum likelihood estimates of $r$ and $\epsilon$ are 0 and 1, respectively; the probability of the data at this point is unity and the process is identifiable. For any $n > 1$, we have the constraint that $\hat{\epsilon} \geq 0$, or

$$\frac{n - e^{rt}}{n-1} \geq 0 \qquad [35]$$

and the upper bound on possible values of $r$ is thus given by $\frac{\log(n)}{t}$. Thus, for a given clade with richness $n$ and age $t$, any arbitrary value of $r$ on $0 < r \leq \frac{\log(n)}{t}$ will yield a valid relative extinction estimate $\epsilon$ for which the probability of the data is equal to equation 17.

For any given multi-clade dataset, consisting of paired age-richness values, researchers can therefore choose a single value of $r$ for all clades (provided it satisfies the inequality above) and, following equation 31, compute clade-specific estimates of the relative extinction fraction $\epsilon$. **Although researchers typically assume that clades vary in $r$, this cannot be justified from the data; it is an assumption**. This decision is no more justifiable than a decision to assume constant $r$ and variable $\epsilon$ across clades; both "models" have identical probability and complexity. However, the biological interpretation of these two approaches is profoundly different.

## 2. Supplementary Note 2: Supplementary methods

**A. Simulation of species richness under the ARR process.** Simulation of species richness under the ARR process is straightforward. As described above, the probability that a process beginning with a single species has exactly $n$ species at time $t$, conditioned on non-extinction, is given by

$$P_{n,t|n\geq 1} = (1-\beta)\beta^{n-1} \quad [36]$$

This is a a shifted geometric distribution with parameter $1-\beta$, where the corresponding geometric density is given by

$$P(k) = (\theta)(1-\theta)^k \quad [37]$$

It follows that we can simulate species richness for the ARR process by sampling from $1 + f_g(1-\beta)$, where $f_g(1-\beta)$ denotes a draw from the geometric distribution with parameter $1-\beta$.

**B. Push of the Past.** The "Push of the Past" (POTP) is a statistical phenomenon whereby clades that diversify under an evolutionary process of high background extinction will appear to have have higher rates of diversification early in their history, even if true diversification rates have been constant through time (5, 6). In effect, POTP is a form of survivorship bias: we only see clades if they survive to the present to be observed (as in ARR studies of extant taxa), and the clades that we see are more likely to have had an apparent *flying start* by chance alone. Even if diversification rates have been invariant through time, such surviving clades will be a nonrandom sample from the total population of diversification processes that existed. The survivors will typically have an excess of lineages early in their history: the clades that have a few extra speciation events when diversity is low will be less likely to go extinct than those that failed to diversify early in their history. Over time, we expect that the estimated clade rate converge on the true rate (a form of regression to the mean). But this phenomenon nonetheless should result in apparent fast rates (e.g., overestimation of the true rate) early in a clade's history. A variant of this phenomenon has also been described for time-calibrated phylogenetic trees (7). Because POTP is expected to generate spuriously high rates for young clades, the analysis of clade-level traits or biogeographic states with respect to ARR rates will be compromised if POTP is the primary source of rate variation among clades.

Nonetheless, we tested whether POTP could explain the apparent decline in rates observed for our focal time series. We simulated "surviving" clades of the same size as the focal clade under a constant-rate birth-death process, but tracking their diversity through time such that realized diversification rates could be computed at any point in time. For each non-extinct clade in our dataset, we first computed the maximum likelihood estimate of $r$ under assumed extinction fractions of $\epsilon = 0.90$ and $\epsilon = 0.98$. We then performed 100,000 simulations under each parameter set, retaining only the set of simulations that yielded an ultimate value of species richness at the end of the simulation that was within 5% of the observed diversity. For example, for the gastropod simulations, we retained all simulated datasets that yielded 37,000 $\pm$ 1,850 species. For each such simulation, we recorded the trajectory of diversity through time. Using this realized trajectory, we then computed the realized rates at a set of equally spaced timepoints for each diversity trajectory. Specifically, for any time $t$, we seek the expected species richness of the process, conditional on the extant richness of the clade at time $T$, or $E[n_t|r, \epsilon, n_T]$. We performed inference under $\epsilon = 0.90$. The POTP effect is strongest when:

- The extinction fraction $\epsilon$ in the true or simulated process is high
- The value of $\epsilon$ used for inference with ARR is low relative to the true value.

Conversely, POTP is much weaker when the the inference model assumes the true value of $\epsilon$ in the generating process. Because researchers frequently conduct analyses with $\epsilon = 0.9$, we used this value for inference. Our analyses of datasets with high ($\epsilon = 0.98$) extinction fractions (and mismatched to the inference model, with $\epsilon = 0.9$) should thus generate a fairly severe POTP.

Our analyses reveal that POTP can lead to time-scaling of rates, consistent with (5). However, the process appears unable to generate the severe time-scaling of rates observed in the paleontological time series we considered (Figure S4). Even under a simulation process with $\epsilon = 0.98$, the observed POTP is only a modest contributor to decline of rates with respect to timescale of measurement. Regardless, POTP is another source of concern for ARR analyses (main text: Figure 5D) and will yield spurious variation in evolutionary rates even when true rates are invariant. We expect that the potential impact of POTP on empirical analyses will be most acute when the estimated range of ARR values across a set of clades is narrow, as in many recent ARR studies (8).

**C. Stability of rates for individual subclades through time.** Even if ARR estimates vary through time, the relationships between rates for contemporaneous clades might nonetheless be stable. In other words, rates might systematically change through time due to time-scaling or other factors, but the relative ordering of ARR rates among contemporaneous clades might be similar. For example, we would want clades inferred to have fast rates at some point in time $t_1$ relative to other clades, to also have fast rates (relative to the same set of clades) at some other time $t_2$. For tests of the relationships between ARR rates and clade-specific traits to be valid, such a relationship must hold in a general sense. Let $\mathbf{X_t}$ denote a vector of clade rates for some set of $k$ clades at time $t$, such that $\mathbf{X_t} = \{r_{1,t}, r_{2,t}, ...r_{k,t}\}$. We expect that $\mathbf{X_t}$ should be correlated with the corresponding vector of ARR rates for the same set of $k$ clades, computed at some other point in time. We therefore have the expectation that $\mathrm{corr}(\mathbf{X_{t_1}}, \mathbf{X_{t_2}}) > 0$.

To test whether subclade rates are indeed correlated through time, we performed a simple analysis of rates computed for order-level clades from one of the best-sampled paleontological timeseries that we considered, the trilobites. To estimate richness for trilobite subclades, we downloaded all Phanerozoic occurrences of Trilobita from the Paleobiology Database www.paleobiodb.org on 2nd January 2020, removing all generically-indeterminate occurrences and all occurences from unlithified or poorly lithified sediments (following (9)). Agnostids and eodiscids were removed from the dataset. Occurrences were sorted into orders Asaphida, Corynexochida, Lichida, Odontopleurida, Phacopida, Proteidae, Ptychopariida and Redlichiida for analysis of subclades. Occurrences were assigned to intervals from the modified time bin scheme of Alroy ((9); modified according to the Geological Timescale 2012; (10)) that was used to analyse our other datasets. We estimated trilobite subclade richness using the Chao richness estimator, making use of incidence frequencies (Chao-2; (11)), as implemented in the R package iNEXT (12). We estimated stem ages for each order as the maximum plausible age of the first occurrence for each order-level taxon in the PBDB data.

For each time slice and trilobite order, we computed the ARR estimated rate assuming $\epsilon = 0.9$ from the stem age and chao-2 estimate of richness at that time. Corresponding rates for each subclade are shown in Figure S5A. For each pair of timepoints $(t_1, t_2)$, we then computed the pairwise correlation between rates for the set of clades that were in existence at each time. Any clades that were present for only one of the timepoints (e.g., they had not yet originated by time $t_1$, or had gone extinct before time $t_2$) were dropped from that comparison. If at least 3 clades were present for both timepoints, we computed the Pearson and Spearman correlations in the corresponding ARR rates. We analyzed these correlations as a function of the lag time between timepoints, as illustrated in Figure 1E in the main text.

Remarkably, there is minimal correlation between ARR rates computed for a set of clades at some time $t_1$ and the corresponding rates for the same clades computed at some other time $t_2$. Across all 67 lags with $n \geq 3$ clades in common, the correlation is, at best, marginally positive (Figure S5). The mean Pearson (Fig. S5B) and Spearman (Fig. S5C) correlations are $\rho = 0.13$ and $\rho = 0.13$, respectively (one-sample t-test: $p = 0.09$ and $p = 0.08$). Even this marginal trend towards significance is driven solely by very short lag times. Repeating these analyses for the set of 51 paired timepoints where the lag time exceeds 20 million years (e.g., $|t_1 - t_2| \geq 20$), we find no correlation between rates. For this subset, mean Pearson and Spearman correlations were 0.03 and 0.01, and corresponding p-values from a one-sample t-test were $p = 0.91$ and $p = 0.69$ respectively ($df = 50$). Thus, at least for trilobites, there is virtually no correlation between subclade rates measured at different timepoints in the overall clade's history.

**D. Comparing absolute prediction error across models.** To supplement the probabilistic model comparisons described in the main text, we tested whether the expected richness under the ARR process has lower absolute error than the predicted richness under the three null models. We compared the deterministic richness predictions for the ARR model against those from each null model. For each pair of timepoints ($t_1$, $t_2$) with corresponding richness values of ($n_1$, $n_2$), prediction error under the "constant" model was computed as $|n_2 - n_1|$. We also included a "random" model, where species richness was assumed to undertake an uncorrelated random walk through time. For this analysis, we drew 100 species richness values from a discrete uniform $(1, N_{MAX})$ distribution, where $N_{MAX}$ is the maximum richness observed in the clade across its entire history. Prediction error was computed as the mean of $|u - n_2|$, where $u$ is the corresponding uniform random deviate. For the *random* model, the expected richness was $1 + \frac{N_{MAX}-1}{2}$, where $N_{MAX}$ is the maximum species richness ever observed in the clade throughout it's history. For the "zero" model (e.g., zero net diversification; $r = 0$, we note that species richness is simply expected to follow a linear model, $n(t) = 1 + \lambda t$ using the maximum likelihood estimator described above. The expected richness at time $t_2$ is then given by $E[n_2] = 1 + (t_2/t_1)(n_1 - 1)$, and the absolute error in the prediction readily computed as $|E[n_2] - n_1|$. For each ($t_1$, $t_2$) pair, we assessed model fit through pairwise comparisons between ARR predictions and each of the three null models. For a given comparison (e.g., ARR and constant) and lag class, we tabulated the proportion of ($t_1$, $t_2$) timepoints where the ARR model fit the data better (e.g., had a lower absolute error) than the focal model. The results in Table S3 and Table S4 factor the data by positive and negative lags. For positive lags, a value of 0.02 for the "constant" model would indicate that the absolute error in the ARR estimate was greater than that predicted by the constant model for 98% of possible timepoints in the relevant lag class. A value of 0.5 implies strict equivalence between models, at least using the binary category of higher or lower absolute prediction error.

Note that a model might yield seemingly low absolute error in comparison to other models, yet the predicted richness might nonetheless be highly unlikely under the parameters of the ARR process. For example, an absolute prediction error of just 10 species appears *small*, but such a number might nonetheless reflect very poor performance of the ARR model if the elapsed time and/or diversification rate are sufficiently small as to make such a change in richness highly improbable.

## 3. Supplementary Note 3: Stem clade ages and justification

**A. Anthozoa.** Molecular phylogenies give a stem age of roughly 617 Ma, based on the split between Octocorallia and Hexacorallia, and at their extremes range from 512-741 Ma (based on 3 molecular studies; (13)). The Precambrian fossil record of metazoans, including Anthozoa is difficult to interpret on account of low skeletonization, but reef corals are known from the early Cambrian. We assigned an estimate of **617 Ma** for this clade. References: (14) and (15).

**B. Articulate brachiopods; Articulata.** A molecular timescale suggests a split of 574 Ma (13) between articulate brachiopods (Rhynchonelliformea) and Lingulata. Numerous taxa are known from the early Cambrian suggesting likely Ediacaran stem age (16, 17). The oldest fossil brachiopods are known from the early Cambrian (18, 19). Sperling et al. (20) conducted a brachiopod-focused molecular clock study that returns an estimated age for the split of Linguliformea and Rhynchionelliformea of 547 Ma (501–587 Ma), but noted that confident assignments of fossils to the stem-lineages of Linguliformea and Rhynchionelliformea did not occur until the Atdabanian (521 Ma), and possibly by the Tommotian (529 Ma), which is well within the error distribution of the estimated divergence time. We therefore used **529** Ma for the stem-group ages of Linguliformea ('Lingulata' of (9)) and Rhynchionelliformea ('Articulata' of (9)). Stem age used: **529** Ma.

**C. Bivalve mollusks: Bivalvia.** Molecular phylogenies give stem age of 538 Ma for Bivalvia (split from Gastropoda) (13). This age is consistent with stem taxa known from the early Cambrian, including *Fordilla* (Cam 2) and *Pojetaia* (Cam 3) (21–23). Vinther et al. (2012, table S4)(24) set bounds on the bivalve-gastropod divergence between 543 and 530 Ma based on fossil occurrences. This provides stem-group ages for both bivalves and gastropods of 530-543 Ma. Given variation in shell composition among some early candidates for stem-group bivalves or gastropods and resulting uncertainties about their affinities (25), we conservatively used **530 Ma** as the stem-group age of Bivalvia and Gastropoda.

**D. Bryozoa.** The oldest stem bryozoan, *Pywackia*, dates to the late Cambrian; U-Pb dates place this at approximately 491 Ma (26). We assigned a stem age of **500** Ma to this clade.

**E. Cephalopoda.** Vinther (27) synthesized molecular and paleontological evidence indicating that unambiguous cephalopod fossils are not known before the Furongian (Late Cambrian) *Plectronoceras cambria* (28, 29). We assigned a stem age of **497** Ma to Cephalopoda based on the age of the base of the Furongian (10).

**F. Chondrichthyes.** Molecular phylogenies constrain this age to approximately 453-497 Ma, with a median of 465 Ma, based on 14 studies collated at timetree.org (13). The fossil record indicates the earliest definite osteichthyans, representing the sister-group of Chondrichthyes, during the late Silurian (423 Ma; (30, 31)) and chondrichthyan-like scales as old as 443 Ma (31, 32). Stem age used: **465** Ma.

**G. Crinoidea.** Problematic fossils, such as *Echmatocrinus*, are known from the Ediacaran and Cambrian but cannot unambiguously be assigned to Crinoidea. Oldest unambiguous Crinoidea occur in the Ordovidian (33). The molecular clock analysis of Peterson et al (34) reports a divergence between Crinoidea and Asterozoa of 505 Ma. The molecular clock analysis of Erwin et al (35) recovers a range of ages for crown-group Echinodermata spanning 501-547 Ma, depending on the status of sponges and the prior set for root age. This provides a stem-group age for Crinoidea, and their fig. 1 implies a stem divergence of 510 Ma. We used a stem age of **510** Ma, as a compromise between lack of fossils in Cambrian but a general tendency of molecular phylogenies to find stem ages greater than 500 Ma.

**H. Echinoidea.** Molecular phylogenies have suggested an early Ordovician stem age for Echinodea. Pisani et al. (2012, p. 31)(36) estimates the echinoid stem-group age as "roughly 475 Ma (95% CI = 501–440)". Crown echinoids generally appear in the Devonian. However, the earliest unambiguous stem echinoid from

the fossil record appears to be *Bromidechinus* at approximately 457 Ma (Burrellian). A recent paper reports strong evidence for a stem-group holothurian from 430 Ma (37). Since holothurians are sister to echinoids, this further places further minimum bound on the stem age for the echinoids. Stem age used: **475** Ma.

**I. Gastropoda.** See notes on Bivalvia (bivalve/gastropod split). We assigned a stem age of **530** Ma for Gastropoda.

**J. Graptoloids.** Upper bound on stem age probably constrained by *Chaunograptus*, from the Burgess Shale (38). Numerous other taxa known from approximately 490 Ma onwards. Stem age used: **510** Ma.

**K. Lingulata.** See notes for articulate brachiopods (Rhynchonelliformea; Articulata). Stem age used: **529** (see revisions to articulate brachiopod stem age)

**L. Ostracoda.** Molecular phylogenies put the split between Ostracoda and Decapoda at 527 Ma, between Ostracoda and Branchiura at 530 Ma, and between Ostracoda and Pentastomida at 530 Ma (13). Earliest generally accepted stem taxon *Kimsella* is known from the early Ordovician (Tremadocian; 485 Ma; (39)), though controversial Cambrian taxa are also known (39). Stem age used: **520** Ma.

**M. Trilobita.** The oldest fossil trilobites are from the Series 2 of the Early Cambrian, at 519 Ma (40, 41). Paterson et al. (41) presented a morphological clock analysis of trilobites and returned an estimated time of origin of 541.3 Ma. Stem age used: **541** Ma.

**N. Macroperforate foraminifera.** Aze et al (2011) (42) note that: "The majority of modern (36 out of 45) and Cenozoic fossil planktonic foraminifera belong to the Superfamily Globigerinacea, thought to be a monophyletic clade that originated in the Lower Maastrichtian, approximately 70 Ma", and indicate that extant macroperforate foraminiferans descend from two species in one genus that crossed the Cretaceous-Paleogene boundary 66 Ma. Stem age used: **70** Ma.

**O. Dinosauria (non-avian).** We used a stem age of **251.9** Ma, corresponding to the start of the Triassic. The oldest definite dinosaur fossils are from the Carnian (middle Triassic) of South Africa (43) and *Nyasaurus*, an older possible dinosaur, is known from the Anisian of Tanzania (c.244 Ma) (44). There is some uncertainty about the maximum plausible age of Dinosauria, but we used the earliest Triassic based on probabilistic stratigraphic-calibration of phylogeny making use only of definite dinosaurian fossils (45).

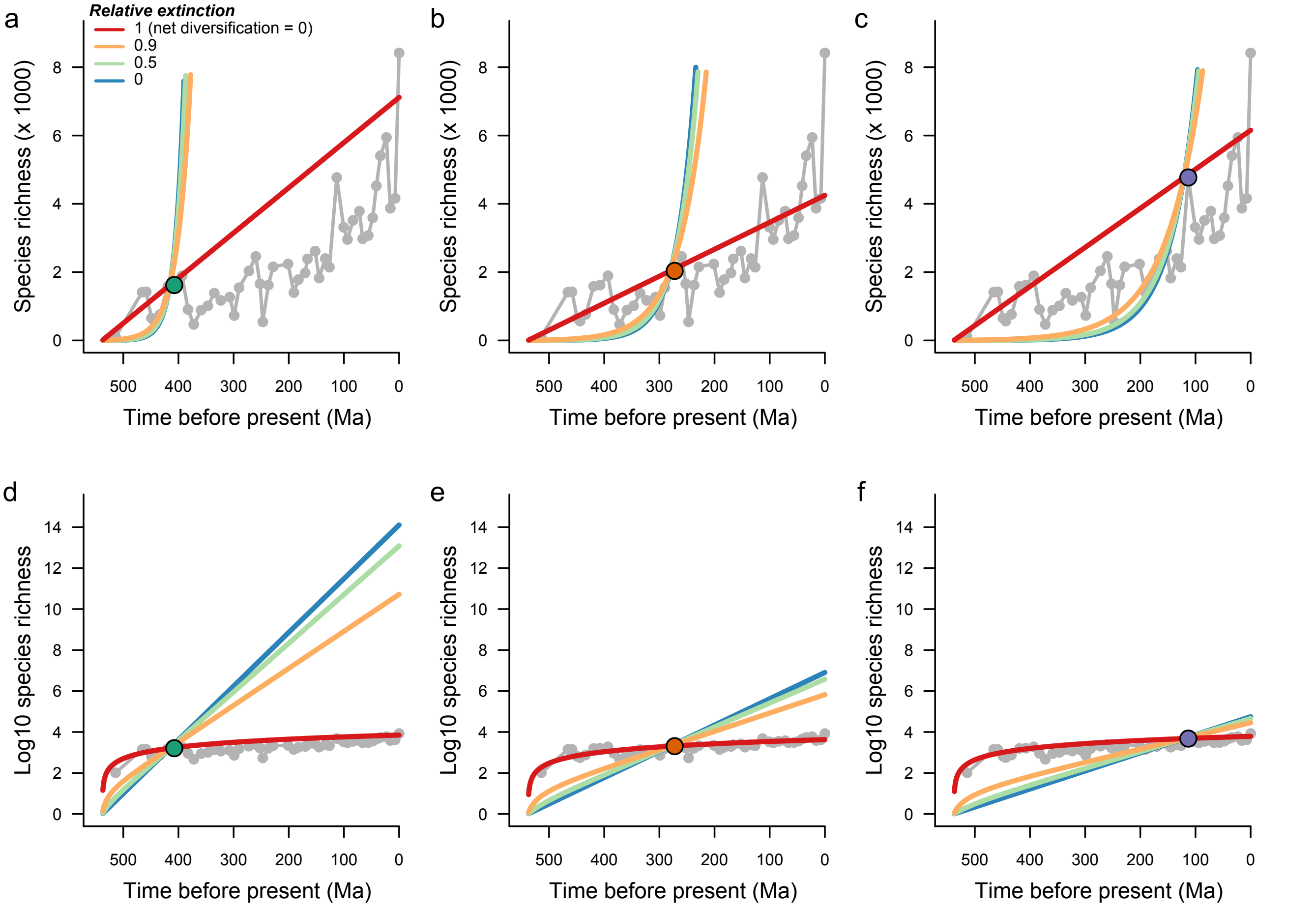


**Fig. S1. ARR estimators predict perfectly the species richness at the focal timepoint used for the calculations, regardless of the underlying relative extinction rate selected for analysis**. The ARR analysis is equivalent to a nonlinear regression with a single datum and forcing the data through the point $n = 1$ (stem clade) or $n = 2$ (crown clade) at time $t = 0$. Each panel (a, b, c) illustrates predictions using the ARR estimate for a single age-richness datapoint across a range of relative extinction rates $\epsilon$. Selected points are identical to those shown in Figure 1 of the main text and can be viewed as the age-richness pair that an observer at a particular point in time would use to compute the ARR estimate (a: Devonian observer; b: Permian observer; c: Cretaceous observer). Complete diversity series for the gastropods (Fig 1) is shown in gray. ARR estimators use only the information from the focal point, along with an assumption about relative extinction ($\epsilon = \mu/\lambda$). The same relationships are shown with species richness on a $\log_{10}$ scale (d, e, f), which more clearly delineates the predicted richness trajectories with different relative extinction. Note that the red line corresponds to the special case with $r = 0$ (e.g., $\epsilon = 1$). Although individual points contain no information with which to validate the assumed extinction fraction, it is clear from visual inspection that that $r = 0$ curve provides a much better fit to this dataset.

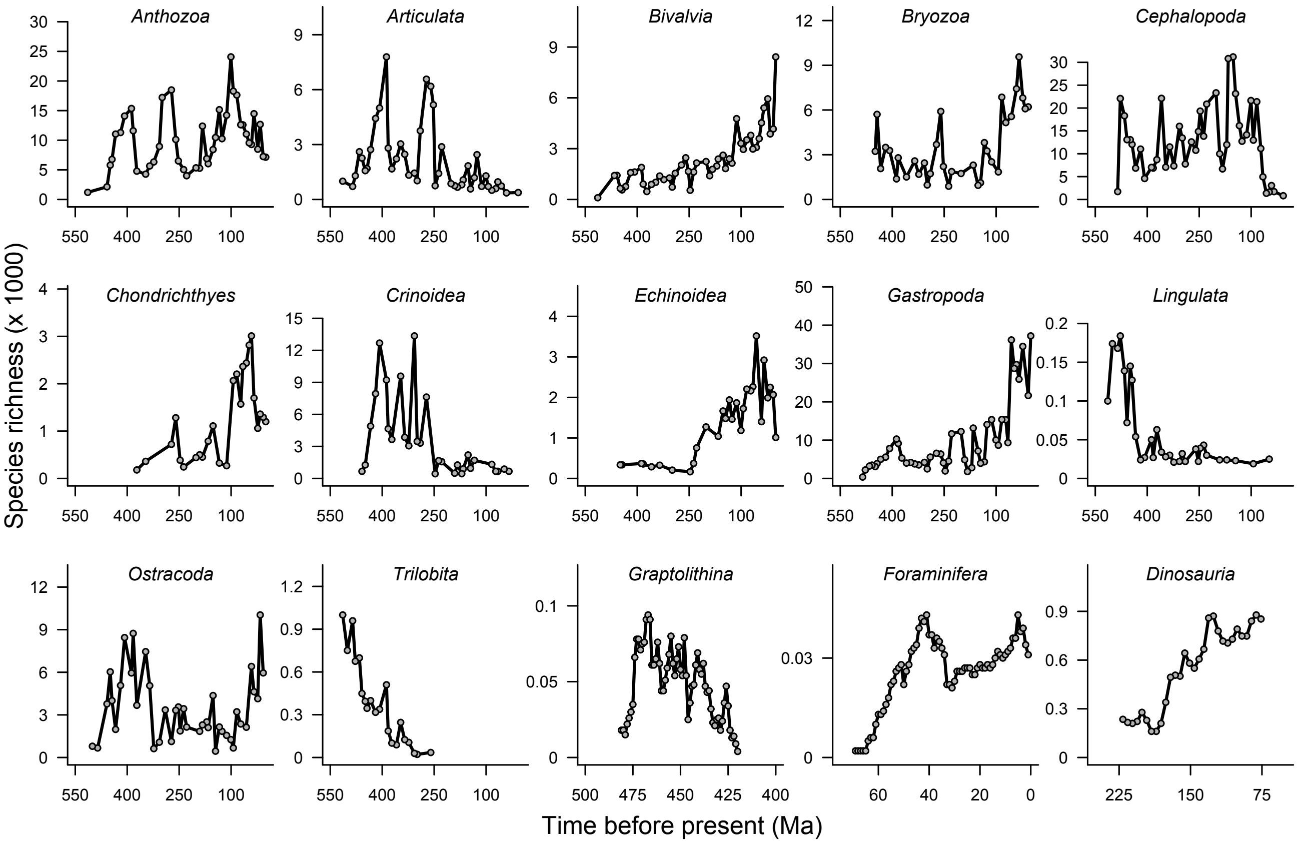


**Fig. S2. Sampling-standardized estimates of species richness through time for 15 clades**. Note that y-axis (species richness) is given in units of 1000 species. Clades span a range of diversity trends, including extinct clades (e.g., Trilobita), declining clades (Lingulata), and rapidly growing clades (Bivalvia, Gastropoda). Dinosauria time series includes no points after the KPg mass extinction. By excluding this interval, we bias our analyses to be more favorable to the ARR estimators.

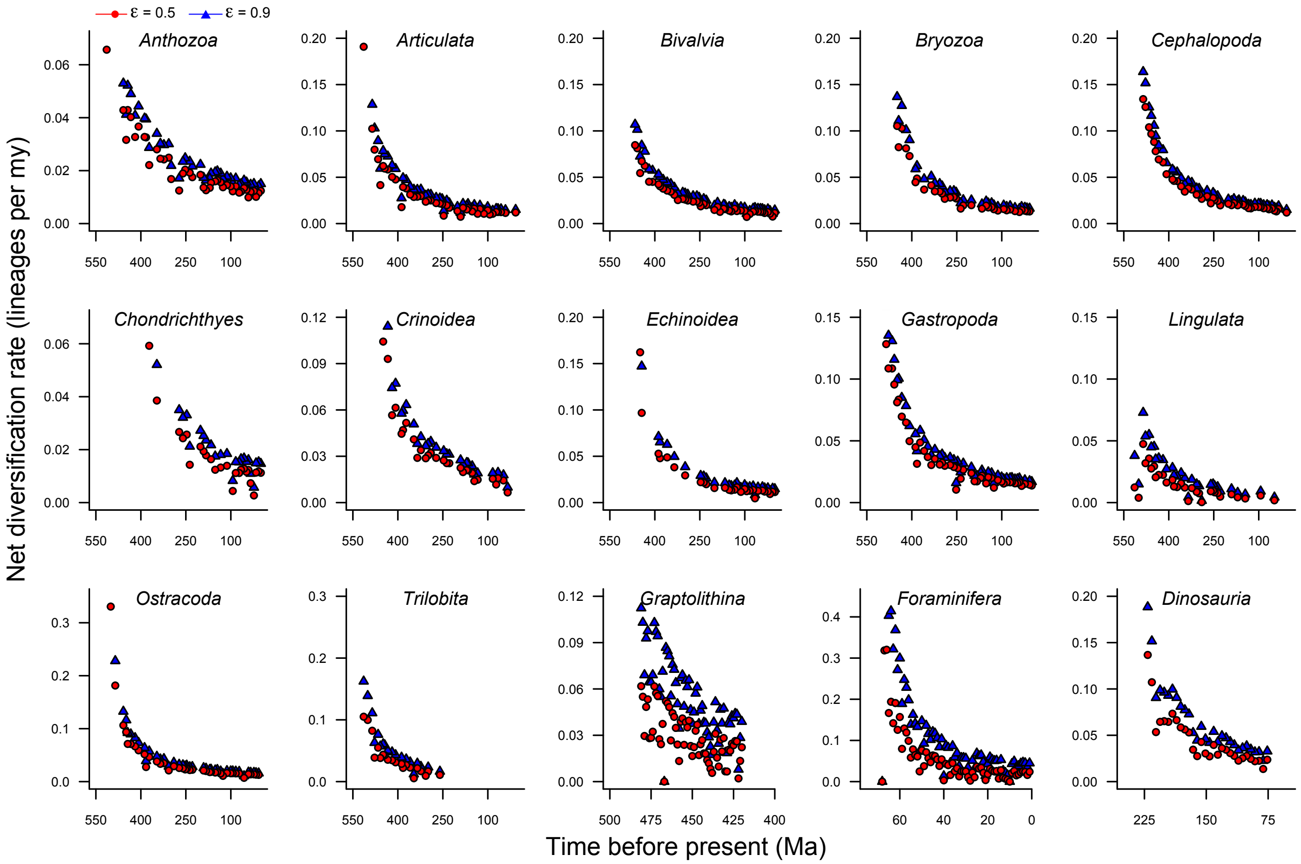


**Fig. S3. Random data yield rate-through-time trajectories that are virtually indistinguishable from those obtained with real fossil diversity trajectories**. Shown are ARR estimates of net diversification rate when species richness data for timepoints in each true diversity curve were sampled from a uniform distribution on the interval (1, $N_{MAX}$), where $N_{MAX}$ is the maximum species diversity ever recorded for the clade. Estimated rates for individual timepoints scale negatively with the duration over which they are computed. Note the similarity between the rate decay phenomenon illustrated in this figure and that shown for the actual diversity data in Figure 2.

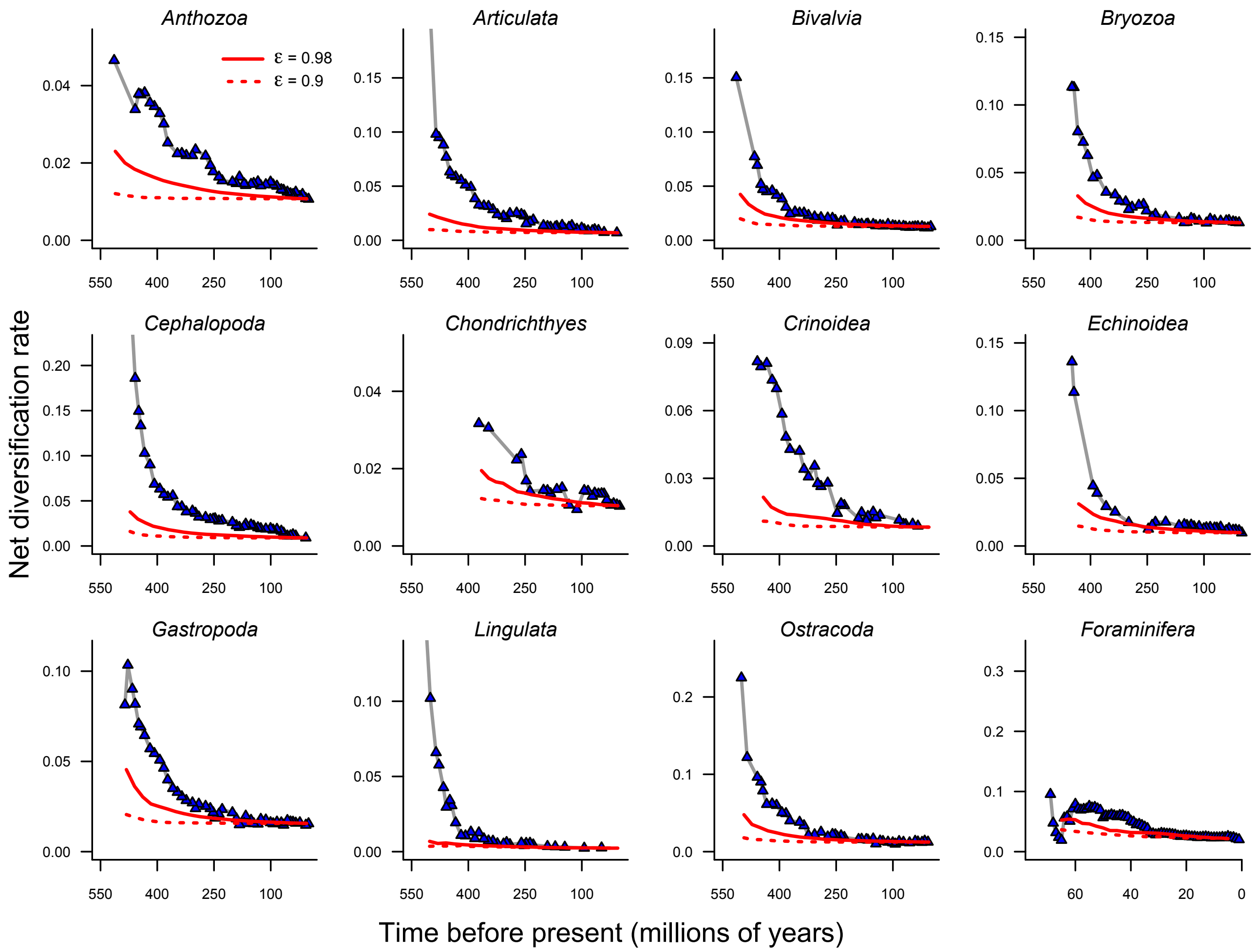


**Fig. S4. Push of the past (POTP) effect for 12 extant clades in the dataset**. ARR rates at different timepoints in each clade's history are are shown with blue triangles and are identical to the $\epsilon = 0.9$ curves shown in Figure 2. Solid and dashed red lines denote the expected ARR trajectory taking POTP into account. Solid line denotes ARR rates for richness trajectories simulated under $\epsilon = 0.98$, a scenario where true relative extinction is much higher than that assumed by the inference model. This mismatch between $\epsilon$ values in the inference model and generating process should result in a more severe POTP. Note that POTP does not yield time-scaling of rates as severe as in the empirical datasets. To the extent that it occurs, POTP is problematic for comparative ARR studies, because it is another source of variation in ARR rates that has no connection to biological process.

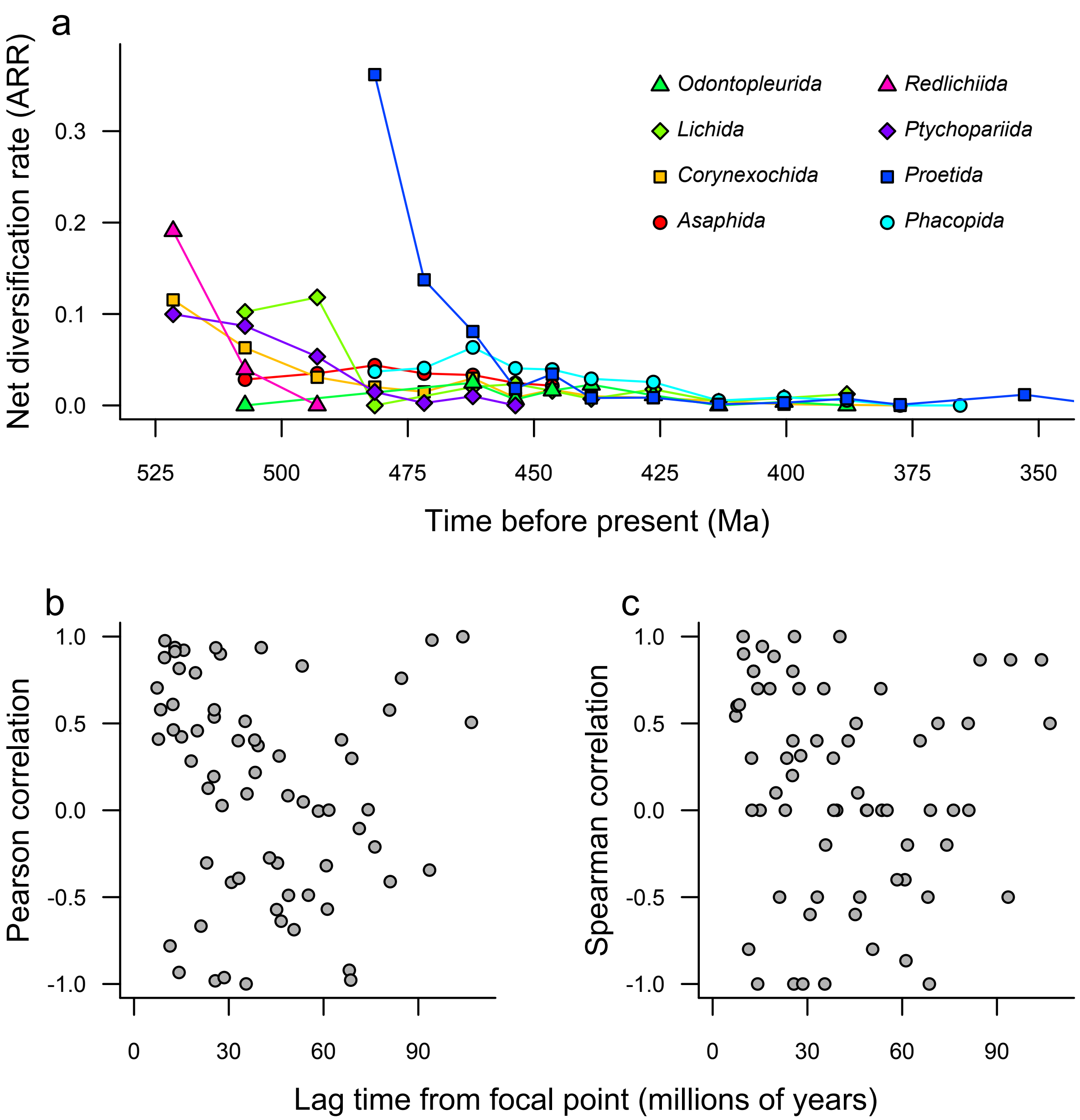


**Fig. S5. Evolutionary rates for trilobite subclades (orders) and their correlation in time**. (a) ARR rates computed with $\epsilon = 0.9$ for 8 orders of trilobites, for the time-slices used in the analyses of the Alroy (9) data. (b) Pairwise Pearson correlation between subclade rates at different timepoints in their history. At best, rates are weakly correlated across short-duration lags ($r = 0.13$, $p \approx 0.09$). However, for lags greater than 20 million years, there is no correlation between subclade rates. c) Corresponding results for Spearman correlation are nearly identical to those for the Pearson correlation (b). See Supplementary Note 2 for further details. Figure 1e in the main text illustrates the calculation of lag times, although in this case, the absolute value of the lag is shown.

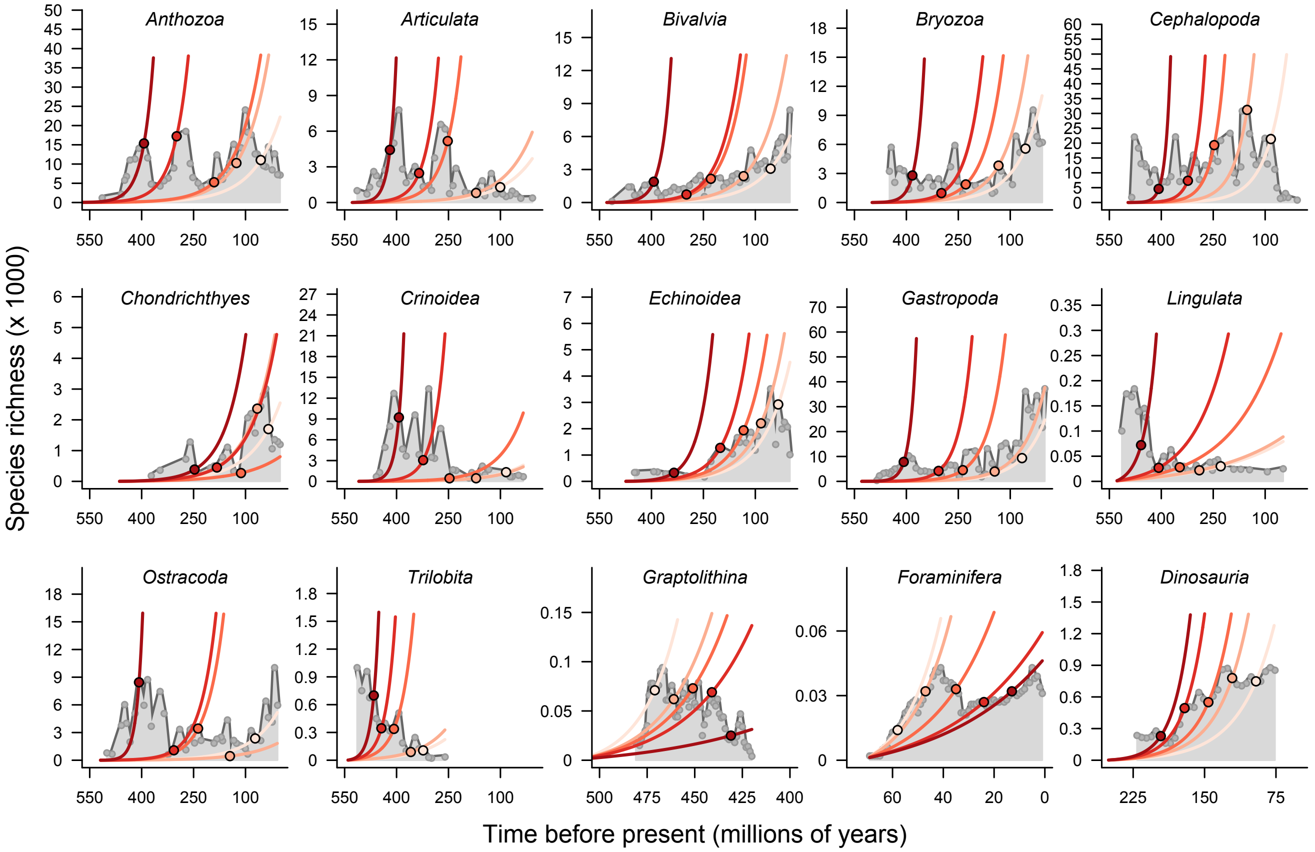


**Fig. S6. Predicted species richness trajectories under ARR estimates for five randomly-selected timepoints for the 15 paleontological time series.** All non-selected points from the full diversity series are shown by gray circles (see Fig. S1). Focal points are shown in shades of red, and corresponding prediction curves pass through each focal point. The ARR estimate thus predicts species richness perfectly only for the timepoint used to calculate the rate, and species richness is generally predicted to increase sharply past the focal timepoint. Relatively extinction rate for this analysis was $\epsilon = 0.9$, and use of other commonly used $\epsilon$ values from the literature (e.g., $\epsilon = 0.5$, $\epsilon = 0$ predicts even more extreme increases in species richness towards the present.

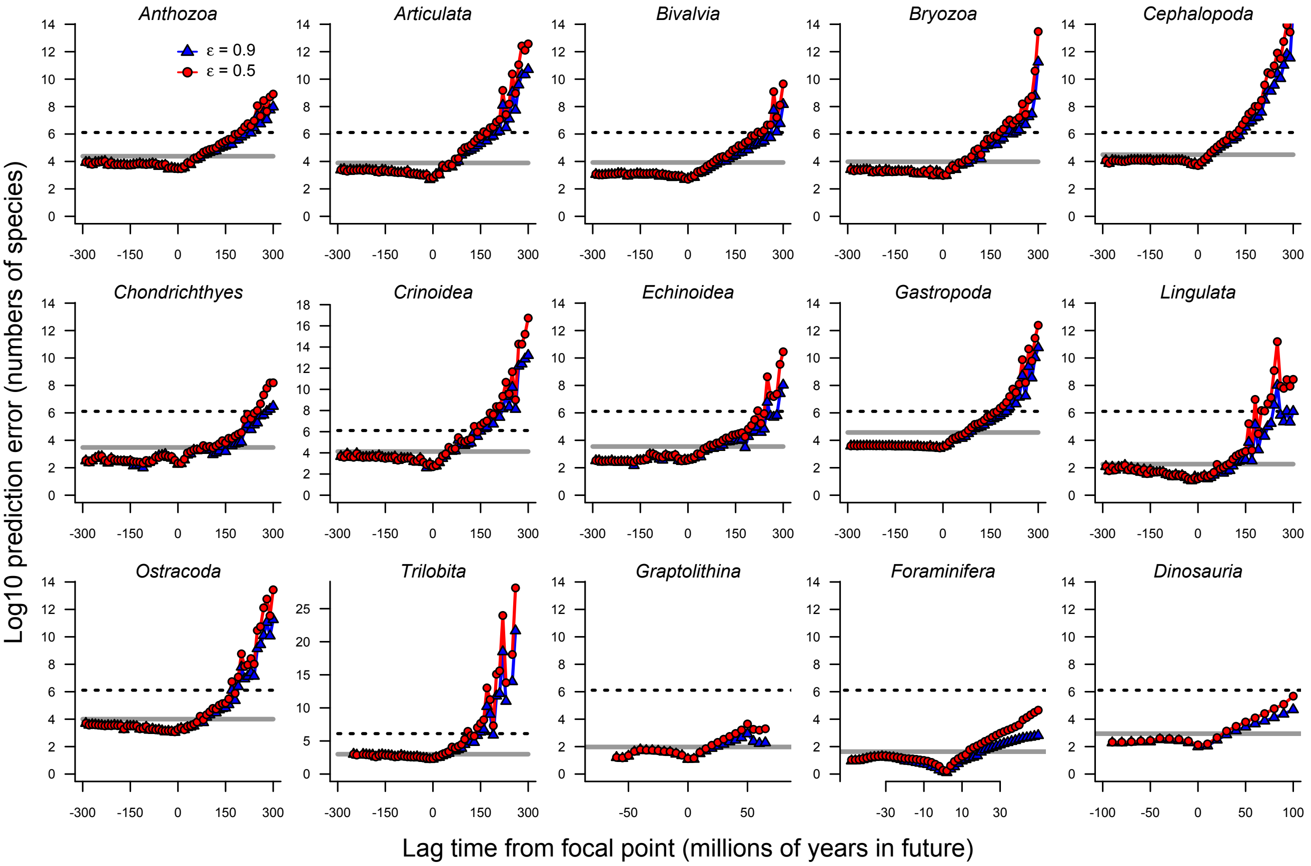


**Fig. S7. Prediction error for ARR estimators ($\epsilon$ = 0.5: red; $\epsilon$ = 0.9: blue) for paleontological diversity-through-time series**. Solid gray line denotes maximum richness observed for each clade at any timepoint; dotted line is number of described species on Earth. Points show median absolute prediction error for all pairs of timepoints that fall within a particular lag bin (e.g., +15 to + 30 my).

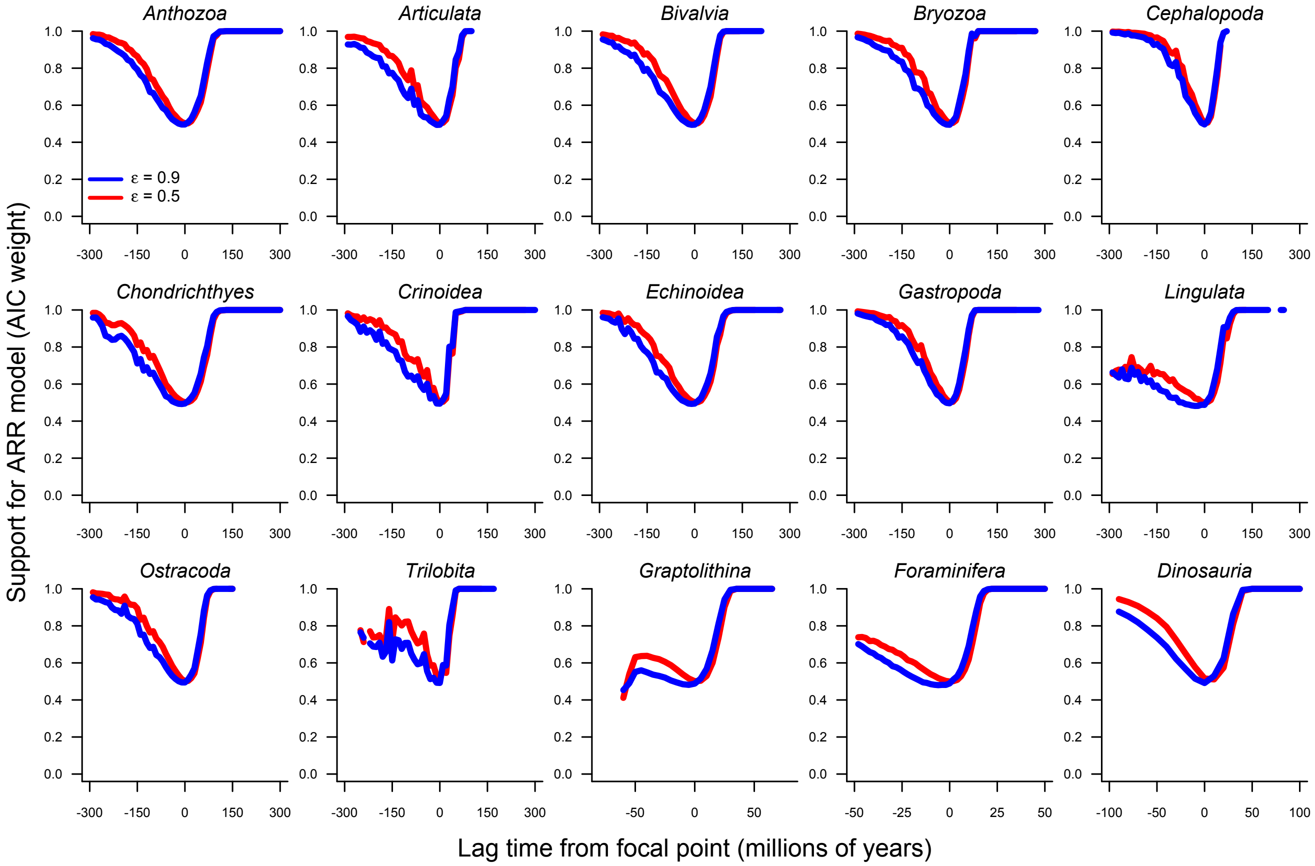


**Fig. S8. Expected AIC weights for ARR process if the ARR process is the true model, relative to constant model**. For each pair of timepoints, species richness at the focal point $t_1$ was used to computed the corresponding ARR rate estimate, $r_1$. This rate $r_1$ was then used to compute the expected richness at another time $t_2$ in the time series. Weights thus reflect the expected evidence favoring the ARR model under a true ARR scenario.

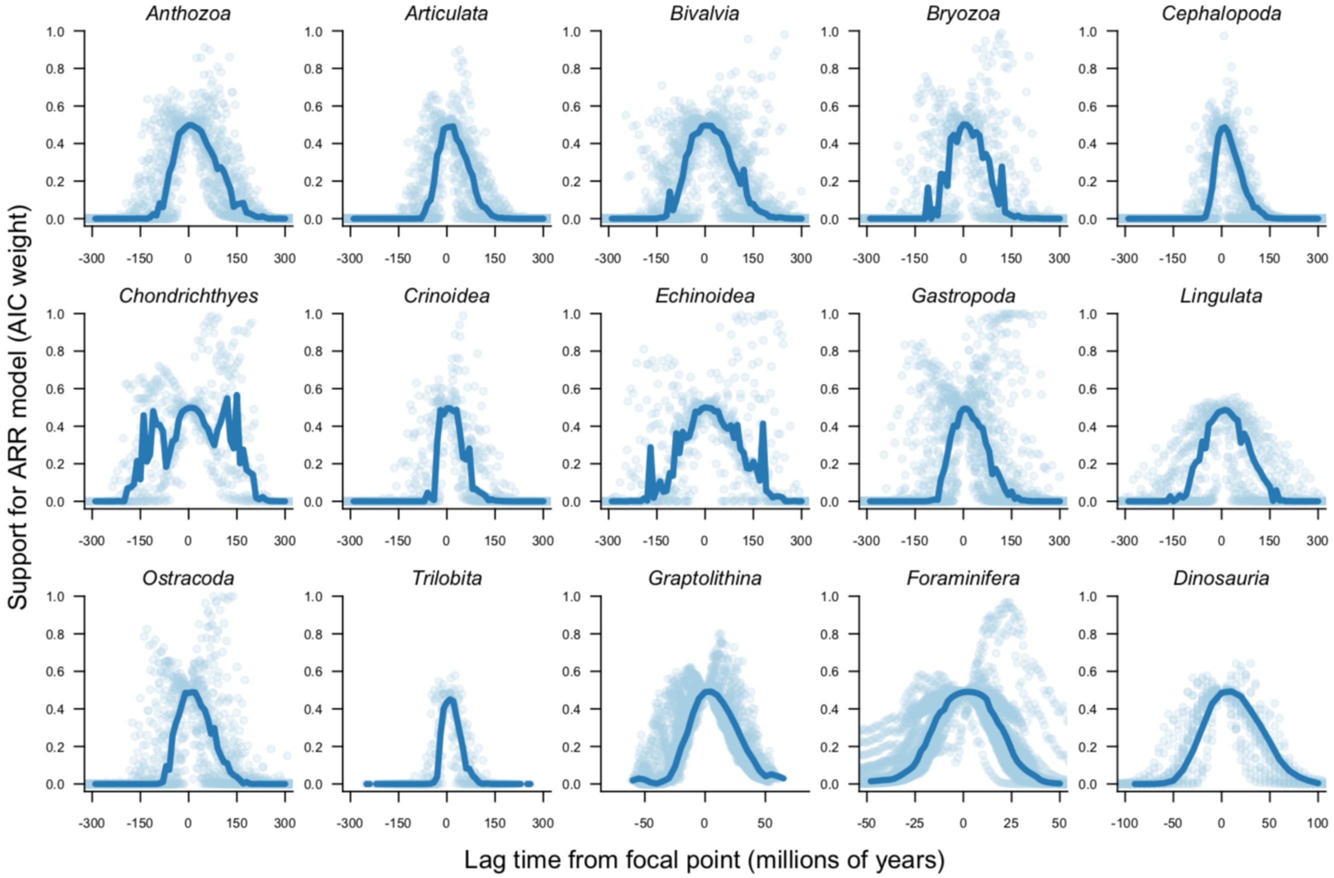


**Fig. S9. Probability (AIC weight) of ARR model relative to zero model ("balanced" speciation extinction; $\lambda = \mu$) as a function of temporal lag**. All pairwise combinations of timepoints are shown for each dataset; red line denotes median weight of ARR model for binned lag times. A value of 0.5 indicates that ARR and random models have equivalent probability; values approaching 1 imply superior performance of the ARR model. For both positive lags (forwards-in-time) and negative lags (backwards-in-time), the ARR model is outperformed by the zero model. Conditioned on survival of the process, the balanced $r = 0$ process predicts a linear increase in species richness through time. The results of this model can also be interpreted non-biologically as a simple linear increase in species diversity through time, for whatever reason. ARR analysis assumes $\epsilon = 0.5$, but results are virtually identical for $\epsilon = 0.9$ (Fig. S9).

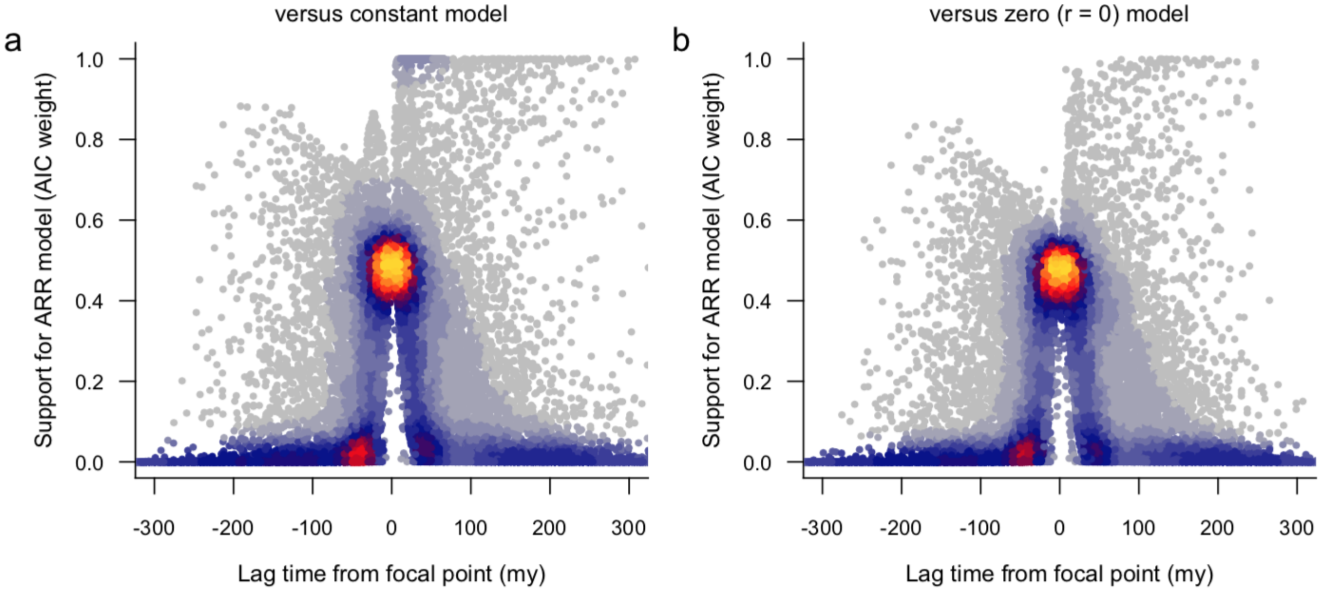


**Fig. S10. Probability of ARR model relative to null models as a function of temporal lag, combined across datasets**. Probability (AIC weight) of ARR model relative to the constant (a) and zero (b) null models, showing all 25740 paired timepoints in the 15 datasets. In general, support for ARR model and null model are approximately equal near the focal point, because species richness is strongly autocorrelated in time (Fig. S1): all models predict that diversity at some time $t_2$ will be similar to the current time $t_1$ as the lag approaches zero. However, outside of this region, support for the ARR model quickly drops to zero. The poor performance of ARR emerges due to underprediction of species richness at negative lags (Fig. 1e), and extreme overprediction at positive lags (Fig. 3).

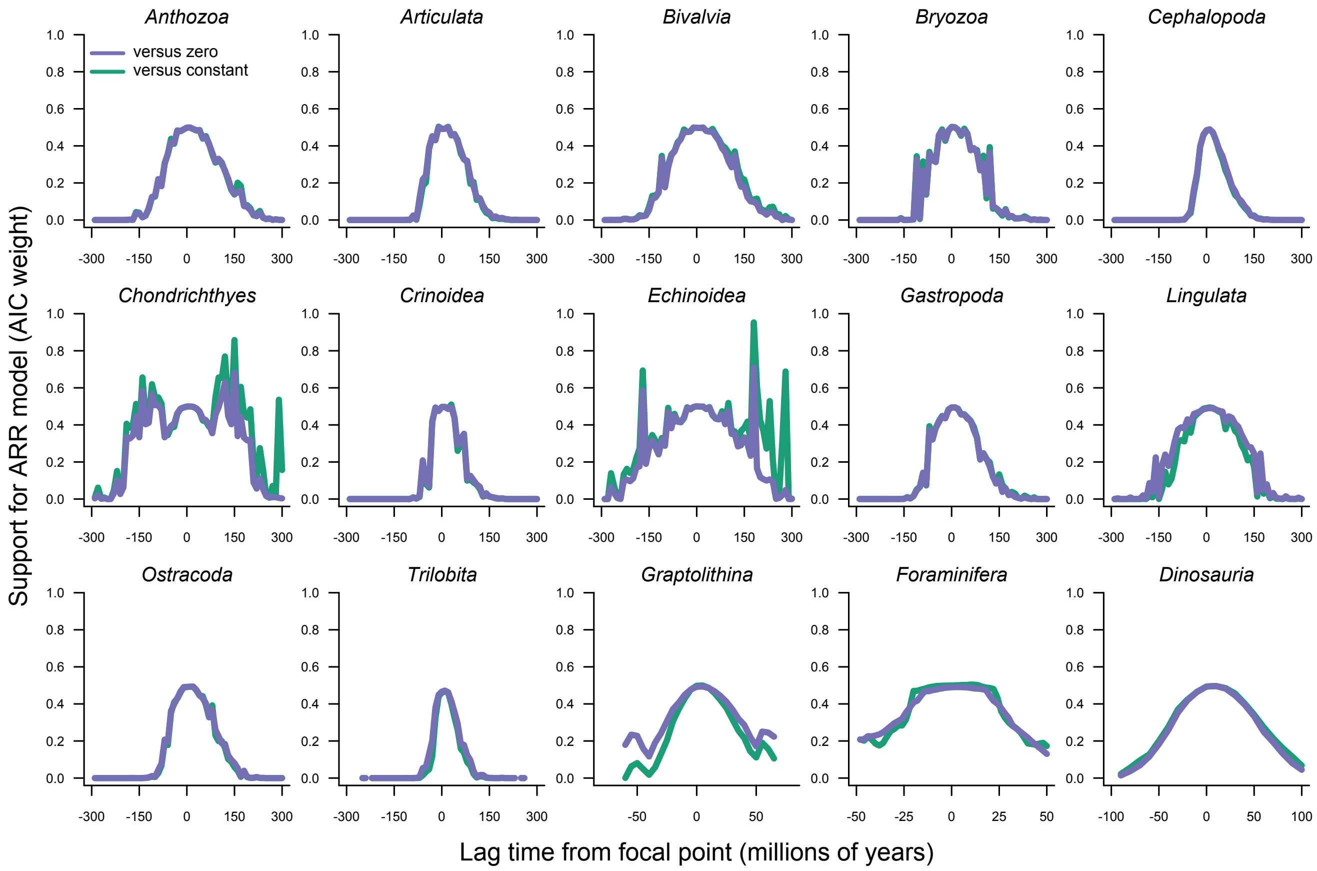


**Fig. S11. Probability of ARR model relative to null models as a function of temporal lag, for each dataset considered individually**. Probabilities (AIC weight) of ARR model relative to two null models (constant, zero); analyses assume a relative extinction rate of $\epsilon = 0.9$. Results are very similar to those shown individually for the three models with $\epsilon = 0.5$ (Fig. 4, Fig. S6, Fig. S7) and indicate superior performance of the null models relative to ARR. In each pairwise model comparison, the relative probability of the ARR model drops to zero for both positive and negative lags.

**Table S1. Summary of paleontological datasets analalyzed in this study**. Valid bins gives the number of timepoints for which an estimate of species richness was available. First and last bins give the age or reference stage of the earliest and latest (most recent) time bin for each series. Recent richness is the number of species inferred to be extant today. Richness multiplier gives the proportionality factor used to estimate species-level species richness at all previous time points. For our purposes, it does not matter that some of the diversity estimates are per time bin (e.g., (9): we simply assume that the ratio of binned diversity values for bins is equal to the ratio of diversities that we would observe for paired timepoints. With this assumption, we can estimate total species richness for a hypothetical time slice from each bin, provided we have an estimate of total diversity for at least one timepoint from the series. Typically, this normalization was performed by equating the most recent time bin (Cz8 or Cz7) with the present-day species richness of the clade. For graptoloids and foraminifera, the original diversity series was given in species-level units, which we accepted at face value. Because richness for Dinosauria and foraminifera were extracted from phylogenies, a set of equally-spaced bins was arbitrarily chosen to be consistent with numbers of bins in other taxa (e.g., in forams, we simply tabulated diversity at 1 my intervals).

| Clade | Source | Valid bins | First bin | Last bin | Stem age | Recent richness | Richness multiplier |
|---|---|---|---|---|---|---|---|
| Anthozoa | (9) | 43 | Cm2 | Cz8 | 617 | 7138 | 301.3 |
| Articulata | (9) | 44 | Cm2 | Cz7 | 529 | 377 | 75.1 |
| Bivalvia | (9) | 47 | Cm2 | Cz8 | 530 | 8418 | 56.9 |
| Bryozoa | (9) | 34 | O4 | Cz7 | 500 | 6208 | 187 |
| Cephalopoda | (9) | 45 | Cm4 | Cz7 | 497 | 812 | 429.6 |
| Chondrichthyes | (9) | 25 | D4 | Cz8 | 465 | 1200 | 90.2 |
| Crinoidea | (9) | 30 | O3 | Cz4 | 510 | 670 | 223.3 |
| Echinoidea | (9) | 29 | O4 | Cz8 | 475 | 1012 | 112.4 |
| Gastropoda | (9) | 47 | Cm4 | Cz8 | 530 | 37201 | 187.7 |
| Lingulata | (9) | 31 | Cm2 | Cz2 | 529 | 25 | 12.5 |
| Ostracoda | (9) | 41 | Cm3 | Cz7 | 520 | 5956 | 223.2 |
| Trilobita | (9) | 21 | Cm2 | P3 | 541.3 | 1000 | 9.45 |
| Dinosauria | (46) | 30 | 221 | 76 | 251.9 | 853 (pre-KPg) | 6.18 |
| Foraminifera | (42) | 69 | 69 | 1 | 70 | 32 | 1 |
| Graptoloids | (47) | - | - | - | 510 | 0 | 1 |

**Table S2. ARR net diversification rates for the first ($r_1$) and last ($r_2$) time bins, for each of the 15 diversity series analyzed**. Rates were computed under the two most widely-used relative extinction rates in the literature ($\epsilon = 0.5$, $\epsilon = 0.9$). *Ratio* gives the ratio of the corresponding rates for each extinction rate. For example, a ratio of 10 implies a 10-fold decrease in the ARR estimated net diversification rate throughout the history of the clade.

| Clade | status | $r_1$ ($\epsilon = 0.5$) | $r_2$ ($\epsilon = 0.5$) | ratio ($r_1/r_2$) | $r_1$ ($\epsilon = 0.9$) | $r_2$ ($\epsilon = 0.9$) | ratio ($r_1/r_2$) |
|---|---|---|---|---|---|---|---|
| Anthozoa | extant | 0.06 | 0.01 | 4.68 | 0.05 | 0.01 | 4.37 |
| Articulata | extant | 0.41 | 0.01 | 41.23 | 0.31 | 0.01 | 43.9 |
| Bivalvia | extant | 0.25 | 0.02 | 15.65 | 0.15 | 0.01 | 11.84 |
| Bryozoa | extant | 0.14 | 0.02 | 8.87 | 0.11 | 0.01 | 8.67 |
| Cephalopoda | extant | 0.58 | 0.01 | 47.49 | 0.44 | 0.01 | 49.35 |
| Chondrichthyes | extant | 0.05 | 0.01 | 3.53 | 0.03 | 0.01 | 3.07 |
| Crinoidea | extant | 0.11 | 0.01 | 9.23 | 0.08 | 0.01 | 9.23 |
| Echinoidea | extant | 0.2 | 0.01 | 15.03 | 0.14 | 0.01 | 13.97 |
| Gastropoda | extant | 0.12 | 0.02 | 7.23 | 0.08 | 0.02 | 6.67 |
| Lingulata | extant | 0.26 | 0.01 | 49.05 | 0.16 | 0 | 62.62 |
| Ostracoda | extant | 0.31 | 0.02 | 19.67 | 0.22 | 0.01 | 18.03 |
| Trilobita | extinct | 0.23 | 0.01 | 22.17 | 0.17 | 0.01 | 32.11 |
| Graptoloids | extinct | 0.08 | 0.01 | 9.76 | 0.03 | 0 | 20.06 |
| Foraminifera | extant | 0.41 | 0.04 | 10.09 | 0.1 | 0.02 | 4.74 |
| Dinosauria (non-avian) | extinct | 0.15 | 0.03 | 4.48 | 0.1 | 0.03 | 4.08 |

**Table S3. Fit of ARR model relative to three null models (constant, random, zero) across all pairs of timepoints, tabulated separately for positive and negative lags**. "Fit" is the proportion of paired timepoints for a given dataset and lag class where the absolute prediction error under the ARR model is lower than for the corresponding null model. A value of 0.05 would indicate that the ARR model performed better than a given alternative for just 5% of paired timepoints. Values greater than 0.5 indicate that the ARR model outperformed the corresponding null model (in terms of absolute prediction error) at least 50% of paired timepoints. Positive and negative lags (after Fig. 1) are denoted by (+) and (-). ARR estimates assumed $\epsilon$ = 0.5; corresponding results for $\epsilon$ = 0.9 are similar and are shown below in Table S4.

| clade | timepoints | vs constant (+) | vs random (+) | vs zero (+) | vs constant (-) | vs random (-) | vs zero (-) |
|---|---|---|---|---|---|---|---|
| Anthozoa | 1806 | 0.07 | 0.11 | 0.07 | 0.35 | 0.56 | 0.18 |
| Articulata | 1892 | 0.05 | 0.13 | 0.05 | 0.17 | 0.6 | 0.08 |
| Bivalvia | 2162 | 0.06 | 0.14 | 0.06 | 0.57 | 0.98 | 0.21 |
| Bryozoa | 1122 | 0.09 | 0.12 | 0.09 | 0.4 | 0.66 | 0.21 |
| Cephalopoda | 1980 | 0.03 | 0.04 | 0.03 | 0.23 | 0.38 | 0.06 |
| Chondrichthyes | 600 | 0.16 | 0.18 | 0.15 | 0.58 | 0.78 | 0.41 |
| Crinoidea | 870 | 0.06 | 0.17 | 0.06 | 0.17 | 0.57 | 0.11 |
| Echinoidea | 812 | 0.09 | 0.14 | 0.09 | 0.62 | 0.79 | 0.31 |
| Gastropoda | 2162 | 0.1 | 0.16 | 0.1 | 0.56 | 0.94 | 0.29 |
| Lingulata | 930 | 0.05 | 0.2 | 0.05 | 0.07 | 0.52 | 0.05 |
| Ostracoda | 1640 | 0.09 | 0.12 | 0.09 | 0.32 | 0.5 | 0.17 |
| Trilobita | 420 | 0.02 | 0.13 | 0.02 | 0.03 | 0.23 | 0.02 |
| Graptoloids | 3782 | 0.13 | 0.19 | 0.12 | 0.22 | 0.41 | 0.15 |
| Foraminifera | 4692 | 0.1 | 0.18 | 0.07 | 0.43 | 0.56 | 0.2 |
| Dinosauria (non-avian) | 870 | 0.04 | 0.1 | 0.03 | 0.49 | 0.6 | 0.14 |
| median | - | 0.07 | 0.14 | 0.07 | 0.35 | 0.57 | 0.17 |

**Table S4. Fit of ARR model relative to three null models (constant, random, linear) across all pairs of timepoints, tabulated separately for positive and negative lags.** "Fit" is the proportion of paired timepoints for a given dataset and lag class where the absolute prediction error under the ARR model is lower than for the corresponding null model. A value of 0.05 would indicate that the ARR model performed better than a given alternative for just 5% of paired timepoints. Values greater than 0.5 indicate that the ARR model outperformed the corresponding null model (in terms of absolute prediction error) at least 50% of paired timepoints. Positive and negative lags (after Fig. 1) are denoted by (+) and (-). Results shown for $\epsilon = 0.9$

| clade | timepoints | vs constant (+) | vs random (+) | vs zero (+) | vs constant (-) | vs random (-) | vs zero (-) |
|---|---|---|---|---|---|---|---|
| Anthozoa | 1806 | 0.1 | 0.13 | 0.09 | 0.36 | 0.58 | 0.19 |
| Articulata | 1892 | 0.06 | 0.18 | 0.05 | 0.18 | 0.61 | 0.09 |
| Bivalvia | 2162 | 0.1 | 0.2 | 0.09 | 0.6 | 0.98 | 0.24 |
| Bryozoa | 1122 | 0.12 | 0.15 | 0.11 | 0.42 | 0.68 | 0.23 |
| Cephalopoda | 1980 | 0.03 | 0.05 | 0.03 | 0.23 | 0.41 | 0.06 |
| Chondrichthyes | 600 | 0.28 | 0.31 | 0.27 | 0.6 | 0.8 | 0.44 |
| Crinoidea | 870 | 0.07 | 0.21 | 0.07 | 0.19 | 0.58 | 0.12 |
| Echinoidea | 812 | 0.16 | 0.2 | 0.15 | 0.65 | 0.83 | 0.35 |
| Gastropoda | 2162 | 0.13 | 0.19 | 0.12 | 0.57 | 0.96 | 0.3 |
| Lingulata | 930 | 0.07 | 0.3 | 0.05 | 0.09 | 0.54 | 0.06 |
| Ostracoda | 1640 | 0.1 | 0.15 | 0.1 | 0.33 | 0.51 | 0.18 |
| Trilobita | 420 | 0.03 | 0.16 | 0.02 | 0.04 | 0.27 | 0.03 |
| Graptoloids | 3782 | 0.19 | 0.26 | 0.16 | 0.25 | 0.47 | 0.18 |
| Foraminifera | 4692 | 0.27 | 0.33 | 0.17 | 0.51 | 0.66 | 0.25 |
| Dinosauria (non-avian) | 870 | 0.07 | 0.16 | 0.06 | 0.53 | 0.67 | 0.18 |
| **median** | - | 0.1 | 0.19 | 0.09 | 0.36 | 0.61 | 0.18 |

## References


1. DG Kendall, On the generalized "birth and death" process. *Annals Math. Stat.* **19**, 1–15 (1948).
2. NTJ Bailey, *The elements of stochastic processes with applications to the natural sciences.* (Wiley), (1964).
3. DM Raup, Mathematical models of cladogenesis. *Paleobiology* **11**, 42–52 (1985).
4. S Magallon, MJ Sanderson, Absolute diversification rates in angiosperms. *Evolution* **55**, 1762–1780 (2001).
5. GE Budd, RP Mann, History is written by the victors: the effect of the push of the past on the fossil record. *Evolution* **72**, 2276–2291 (2018).
6. S Nee, RM May, PH Harvey, The reconstructed evolutionary process. *Philos. Transactions Royal Soc. B: Biol. Sci.* **344**, 305–311 (1994).
7. AB Phillimore, TD Price, Density-dependent cladogenesis in birds. *PLoS Biol.* **6**, e71 (2008).
8. JJ Wiens, Faster diversification on land than sea helps explain global biodiversity patterns among habitats and animal phyla. *Ecol. Lett.* **18**, 1234–1241 (2015).
9. J Alroy, The shifting balance of diversity among major marine animal groups. *Science* **329**, 1191–1194 (2010).
10. FM Gradstein, JG Ogg, MD Schmitz, GM Ogg, *The Geological Timescale.* (Elsevier), (2012).
11. A Chao, Estimating population size for capture-recapture data with unequal catchibility. *Biometrics* **43**, 783–791 (1987).
12. TC Hsieh, KH Ma, A Chao, *iNEXT: Interpolation and Extrapolation for Species Diversity*, (2020) R package version 2.0.20.
13. S Kumar, G Stecher, M Suleski, SB Hedges, Timetree: a resource for timelines, timetrees, and divergence times. *Mol. Biol. Evol.* **34**, 1812–1819 (2017).
14. MJ Hicks, A new genus of early cambrian coral in esmeralda county, southwestern nevada. *J. Paleontol.* **80**, 609–615 (2006).
15. M Fuller, R Jenkins, Reef corals from the lower cambrian of the flinders ranges, south australia. *Paleontology* **50**, 961–980 (2007).
16. DAT Harper, PL E., HL E., Brachiopods: origin and early history. *Palaeontology* **56**, 609–631 (2017).
17. SJ Carlson, The evolution of brachiopoda. *Annu. Rev. Earth Planet. Sci.* **44**, 409–438 (2016).
18. GT Ushantinskaya, Origin and dispersal of the earliest brachiopods. *Paleontol. J.* **42**, 776–791 (2008).
19. Z Zhang, SP Robson, E C., S D., Early cambrian radiation of brachiopods: A perspective from south china. *Gondwana Res.* **14**, 241–254 (2008).
20. EA Sperling, D Pisani, KJ Peterson, Molecular paleobiological insights into the origin of the brachiopoda. *Evol. Dev.* **13**, 290–303 (2011).
21. MJ Vendrasco, AG Checa, A Kouchinsky, Shell microstructure of the early bivalve *Pojetaia* and the independent origin of nacre within the mollusca. *Palaeontology* **54**, 825–850 (2011).
22. O Elicki, S Gursu, First record of *Pojetaia runnegari* jell, 1980 and fordilla barrande, 1881 from the middle east (taurus mountains, turkey) and critical review of cambrian bivalves. *Palaontologische Zeitschrift* **83**, 267–291 (2009).
23. O Elicki, Lower cambrian carbonates from eastern germany: Palaeontology, stratigraphy and palaeogeography. *Neues Jahrbuch fur Geol. und Palaontologie* **191**, 69–93 (1994).
24. J Vinther, EA Sperling, DEG Briggs, KJ Peterson, A molecular palaeobiological hypothesis for the origin of aplacophoran molluscs and their derivation from chiton-like ancestors. *Proc. R. Soc. B* **279**, 1259 – 1268 (2012).
25. L Li, X Zhang, H Yun, G Li, Complex hierarchical microstructures of cambrian mollusk pelagiella: insight into early biomineralization and evolution. *Sci. Reports* **7**, 1935 (2017).
26. Landing, Distinguishing earth's oldest known bryozoan (*Pywackia*, late cambrian) from pennatulacean octocorals (mesozoic–recent). *J Paleontol.* **89**, 215–317 (2015).
27. J Vinther, The origins of mollusks. *Palaeontology* **58**, 19–34 (2015).

28. CE Walcott, Cambrian faunas of china. *Proc. US Natl. Mus.* **29**, 1–106 (1905).
29. E Landing, B Kröger, The oldest cephalopods from east laurentia. *J. Paleontol.* **83**, 123–127 (2009).
30. M Zhu, W Zhao, L Jia, T Qiao, Q Qu, The oldest articulated osteichthyan reveals mosaic gnathostome characters. *Nature* **458**, 469–474 (2009).
31. MD Brazeau, M Friedman, The origin and early phylogenetic history of jawed vertebrates. *Nature* **520**, 490–497 (2015).
32. V Karatajute-Talimaa, N Predtechenskyj, The distribution of the vertebrates in the late ordovician and early silurian palaeobasins of the siberian platform. *Bull. Mus. Natl Hist. Nat.* **4**, 39–55 (1995).
33. DJ Erwin, Early phylogeny and subclass division of the crinoidea (phylum echinodermata). *J. Paleontol.* **72**, 499–510 (1998).
34. KJ Peterson, JA Cotton, JG Gehling, D Pisani, The ediacaran emergence of bilaterians: congruence between the genetic and the geological fossil records. *Phil. Trans. R. Soc. B.* **363**, 1435–1443 (2008).
35. DJ Erwin, et al., The cambrian conundrum: Early divergence and later ecological success in the early history of animals. *Science* **334**, 1091–1097 (2011).
36. D Pisani, R Feuda, KJ Peterson, AB Smith, Resolving phylogenetic signal from noise when divergence is rapid: A new look at the old problem of echinoderm class relationships. *Mol. Phylo. Evol.* **62**, 27–34 (2012).
37. IA Rahman, et al., A new ophiocistioid with soft-tissue preservation from the silurian herefordshire lagerstatte, and the evolution of the holothurian body plan. *Proc. R. Soc. B* **62**, doi.org/10.1098/rspb.2018.2792 (2019).
38. JB Caron, DA Jackson, Paleoecology of the greater phyllopod bed community, burgess shale. *Palaeogeogr. Palaeoclimatol. Palaeoecol.* **258**, 222–226 (2008).
39. M Williams, et al., The earliest ostracods: the geological evidence. *Senckenbergiana Lethaea* **88**, 11–21 (520).
40. R Fortey, RM Owens, *Treatise on Invertebrate Paleontology (Arthropoda 1,Trilobita, Revised)*, ed. RL Kaesler. (University of Kansas Press, Lawrence KS), (1997).
41. JR Paterson, GD Edgecombe, MSY Lee, Trilobite evolutionary rates constrain the duration of the cambrian explosion. *Proc. Natl. Acad. Sci. U.S.A.* **10**, 4394–4399 (2019).
42. T Aze, et al., A phylogeny of cenozoic macroperforate planktonic foraminifera from fossil data. *Biol. Rev.* **86**, 900–927 (2011).
43. MC Langer, MD Ezcurra, JS Bittencourt, FE Novas, The origin and early evolution of dinosaurs. *Biol. Rev.* **85**, 55–110 (2010).
44. SJ Nesbitt, PM Barrett, S Werning, CA Sidor, AJ Charig, The oldest dinosaur? a middle triassic dinosauriform from tanzania. *Biol. Lett.* **9**, 20120949 (2013).
45. GT Lloyd, DW Bapst, F M., DK E., Probabilistic divergence time estimation without branch lengths: dating the origins of dinosaurs, avian flight and crown birds. *Biol. Lett.* **12**, 20160609 (2016).
46. RBJ Benson, et al., Rates of dinosaur body mass evolution indicate 170 million years of sustained ecological innovation on the avian stem lineage. *PLOS Biol.* **12**, e1001896 (2014).
47. M Foote, RA Cooper, JS Crampton, PM Sadler, Diversity-dependent evolutionary rates in early paleozoic zooplankton. *Proc. R. Soc. B.* **285**, 20180122 (2018).
