# Supplementary material for: Ecological and biogeographic drivers of biodiversity cannot be resolved using clade age-richness data
Source: Nat Commun. 2021 May 19;12:2945. doi: 10.1038/s41467-021-23307-5 (PMC8134473; doi:10.1038/s41467-021-23307-5)
Supplement: Supplementary file 3 — Reporting Summary [file 41467_2021_23307_MOESM3_ESM.pdf]

nature research

Corresponding author(s): Daniel L. Rabosky

Last updated by author(s): Apr 20, 2021

Reporting Summary

Nature Research wishes to improve the reproducibility of the work that we publish. This form provides structure for consistency and transparency in reporting. For further information on Nature Research policies, see our Editorial Policies and the Editorial Policy Checklist.

Statistics

For all statistical analyses, confirm that the following items are present in the figure legend, table legend, main text, or Methods section.

n/a	Confirmed	
☐	☒	The exact sample size (*n*) for each experimental group/condition, given as a discrete number and unit of measurement
☒	☐	A statement on whether measurements were taken from distinct samples or whether the same sample was measured repeatedly
☒	☐	The statistical test(s) used AND whether they are one- or two-sided<br>*Only common tests should be described solely by name; describe more complex techniques in the Methods section.*
☒	☐	A description of all covariates tested
☐	☒	A description of any assumptions or corrections, such as tests of normality and adjustment for multiple comparisons
☐	☒	A full description of the statistical parameters including central tendency (e.g. means) or other basic estimates (e.g. regression coefficient) AND variation (e.g. standard deviation) or associated estimates of uncertainty (e.g. confidence intervals)
☐	☒	For null hypothesis testing, the test statistic (e.g. F, t, r) with confidence intervals, effect sizes, degrees of freedom and P value noted<br>*Give P values as exact values whenever suitable.*
☒	☐	For Bayesian analysis, information on the choice of priors and Markov chain Monte Carlo settings
☒	☐	For hierarchical and complex designs, identification of the appropriate level for tests and full reporting of outcomes
☐	☒	Estimates of effect sizes (e.g. Cohen's *d*, Pearson's *r*), indicating how they were calculated

Our web collection on statistics for biologists contains articles on many of the points above.

Software and code

Policy information about availability of computer code

Data collection	No software was used in collection of any data
Data analysis	Data analysis used custom scripts in the R statistical/programming language, R version 3.7.3. All scripts used for data analysis and figure production are included in the Dryad data package and are publicly available at doi: https://doi.org/10.5061/dryad.qz612jmfb

For manuscripts utilizing custom algorithms or software that are central to the research but not yet described in published literature, software must be made available to editors and reviewers. We strongly encourage code deposition in a community repository (e.g. GitHub). See the Nature Research guidelines for submitting code & software for further information.

Data

Policy information about availability of data

All manuscripts must include a data availability statement. This statement should provide the following information, where applicable:

- Accession codes, unique identifiers, or web links for publicly available datasets
- A list of figures that have associated raw data
- A description of any restrictions on data availability

All data analyzed in this study are publicly available. Data for macroperforate foraminifera are available at https://onlinelibrary.wiley.com/doi/full/10.1111/j.1469-185X.2011.00178.x. Dinosaur and graptoloid data were downloaded from the Dryad digital repository at https://datadryad.org/stash/dataset/doi:10.5061/dryad.gr1qp and https://datadryad.org/stash/dataset/doi:10.5061/dryad.fq7h2, respectively. Raw occurrence data used to generate the diversity curves for all other clades are available through the Paleobiology Database (https://paleobiodb.org). Compiled datasets, including fossil diversity time series and associated clade ages, are available as part of the data package that accompanies this article through the Dryad digital data repository (doi: https://doi.org/10.5061/dryad.qz612jmfb).

Field-specific reporting

Please select the one below that is the best fit for your research. If you are not sure, read the appropriate sections before making your selection.

- [ ] Life sciences
- [ ] Behavioural & social sciences
- [x] Ecological, evolutionary & environmental sciences

For a reference copy of the document with all sections, see nature.com/documents/nr-reporting-summary-flat.pdf

Ecological, evolutionary & environmental sciences study design

All studies must disclose on these points even when the disclosure is negative.

Study description	Theoretical and empirical investigation of a widely-used macroevolutionary rate metric. We mathematically assess the theoretical information content of the rate estimator. We then use publicly available data from the fossil record to test whether the rate metric provides accurate estimates of evolutionary rates.
Research sample	We analyzed paleodiversity data from 15 groups of organisms, ranging from marine plankton to dinosaurs. Each dataset was a previously-published "diversity-through-time" trajectory for different group. Data for macroperforate foraminifera are available at https://onlinelibrary.wiley.com/doi/full/10.1111/j.1469-185X.2011.00178.x. Dinosaur and graptoloid data were downloaded from the Dryad digital repository at https://datadryad.org/stash/dataset/doi:10.5061/dryad.gr1qp and https://datadryad.org/stash/dataset/doi:10.5061/dryad.fq7h2, respectively. Raw occurrence data used to generate the diversity curves for all other clades are available through the Paleobiology Database (https://paleobiodb.org).
Sampling strategy	We identified recently-published diversity-through-time datasets for the fossil record that were characterized by high statistical rigor, e.g., use of sampling standardization methods such as SQS. We also required that each dataset include a sufficient number of sampled timepoints (> 25). Relatively few datasets met these critera, leaving us with the 15 that we analyzed.
Data collection	Both authors contributed to the downloading of publicly available source data analyzed here, but no new data were collected.
Timing and spatial scale	Each paleo time series consists of a range of diversity samples at different points in time. In general, we were limited by the temporal resolution and sampling of the original studies.
Data exclusions	No data were excluded from any analyses.
Reproducibility	We performed mathematical calculations several ways to verify that any automated calculations in our computer code were returning the correct values. All such validation indicated that our calculations were correct.
Randomization	Randomization is not relevant to our study as there is no control or treatment associated with the study design.
Blinding	No blinding was used. Blinding was not relevant, because the data were empirical time-series from the fossil record and we were not performing comparisons across the series. In addition, there were no experimental groups for which it would have been possible to blind the investigators.

Did the study involve field work? [ ] Yes [x] No

Reporting for specific materials, systems and methods

We require information from authors about some types of materials, experimental systems and methods used in many studies. Here, indicate whether each material, system or method listed is relevant to your study. If you are not sure if a list item applies to your research, read the appropriate section before selecting a response.

Materials & experimental systems

n/a	Involved in the study	
x		Antibodies
x		Eukaryotic cell lines
	x	Palaeontology and archaeology
x		Animals and other organisms
x		Human research participants
x		Clinical data
x		Dual use research of concern

Methods

n/a	Involved in the study	
x		ChIP-seq
x		Flow cytometry
x		MRI-based neuroimaging

Palaeontology and Archaeology

Specimen provenance	All data analyzed in the study is digital paleobiological time-series data.
Specimen deposition	N/A
Dating methods	N/A

☐ Tick this box to confirm that the raw and calibrated dates are available in the paper or in Supplementary Information.

Ethics oversight	No ethical approval required as study used digital data

Note that full information on the approval of the study protocol must also be provided in the manuscript.
